# Machine learning for immune biomarkers in severe mental illness: a systematic review

**DOI:** 10.1016/j.nsa.2026.107003

**Published:** 2026-04-21

**Authors:** Rayan Slatni, Federica Colombo, Paolo Enrico, Igor Branchi, Livia De Picker, Benedetta Vai

**Affiliations:** a Collaborative Antwerp Psychiatric Research Institute (CAPRI), Faculty of Medicine and Health Sciences, University of Antwerp, Antwerp, Belgium; b Scientific Initiative of Neuropsychiatric and Psychopharmacological Studies (SINAPS), University Psychiatric Centre Campus Duffel, Duffel, Belgium; c Psychiatry and Clinical Psychobiology Unit, Division of Neuroscience, IRCCS Ospedale San Raffaele, Milano, Italy; d Department of Neurosciences and Mental Health, Fondazione IRCCS Ca’ Granda Ospedale Maggiore Policlinico, Italy; e Center for Behavioral Sciences and Mental Health Istituto Superiore di Sanità, Viale Regina Elena, 299, 00161, Rome, Italy; f University Vita-Salute San Raffaele, Milano, Italy

**Keywords:** Severe mental illness, Immunopsychiatry, Computational psychiatry, Machine learning, Immune-biomarkers

## Abstract

The integration of machine learning (ML) approaches with immune biomarker research may facilitate the identification of candidate markers for achieving personalized medicine approaches in severe mental illnesses (SMI). This systematic review synthesizes the available evidence on ML algorithms applied to immune biomarkers in major depressive (MDD), bipolar (BD) and schizophrenic spectrum disorders (SZ), examining their performance across different clinical uses including diagnostic, prediction, monitoring, prognostic categories, in accordance with the Food and Drug Administration - Biomarker, EndpointS, and other Tools (FDA BEST) framework. We performed a PRISMA-compliant systematic search of PubMed, Web of Science, Scopus and PsycINFO databases until 14 July 2025, including 43 eligible studies with a total sample of 11,556 participants, 8339 with SMI (3228 MDD, 2614 BD and 2497 SZs) and 3217 healthy controls. We systematically described population, ML input data (including blood collection conditions, pre-processing steps, sample type, laboratory assay, missing data, and multimodality), and algorithms (supervised versus unsupervised models, feature selection, validation strategy, outcomes, and performance metrics). Overall, ML models showed moderate to high but heterogeneous performance. Diagnostic applications were the most common (AUC = 0.650–0.990), though predictive, monitoring, and prognostic uses were underrepresented and more variable. Across disorders, pro-inflammatory markers (IL-6, IL-8, TNF-α, IFN-γ, CRP) and IL-10 emerged most consistently, and data-driven approaches suggested shared immune subtypes beyond categorical diagnoses. However, substantial methodological and biological heterogeneity was observed, including inconsistent handling of missing data, limited external validation, and variable feature selection. Immunology-specific sources of variability (such as fasting status, circadian rhythms, and measurement batch effects) were rarely addressed, and the long-term stability of immune-based ML signatures remains largely unexplored. These gaps currently limit clinical translation and underscore the need for standardized protocols and more rigorous ML pipelines.

## Highlights

• FDA BEST-guided machine learning review on immune markers in severe mental illness.
• Diagnostic models showed moderate-to-high performance (AUC 0.65-0.99).
• Predictive, monitoring and prognostic uses showed more variable performance.
• Methodological heterogeneity limits clinical translation of machine learning models.

## 1 Introduction

Severe mental illnesses (SMI), including bipolar disorder (BD), major depressive disorder (MDD), and schizophrenia spectrum disorders (SZ), including schizophrenia and other psychotic disorders, rank among the world's leading causes of disability, with substantial costs on healthcare systems (GBD, 2021 Diseases and Injuries Collaborators, 2024; Jin and Mosweu, 2017; Greenberg et al., 2021; Dembek et al., 2023). Despite their significant burden, around one third of patients experience inadequate symptom relief or treatment resistance (McIntyre et al., 2023; Kennedy et al., 2014). This therapeutic gap is partly due to our incomplete understanding of the biological mechanisms underlying these conditions, which hindered the development of new therapeutic targets and personalized treatment approaches (Paul and Potter, 2024). The past decade has seen rapid progress in immunopsychiatry, revealing a central role of the immune system across diverse psychiatric disorders. Dysregulation of the inflammatory response system has been described both in mood disorders and SZ, characterized by central and peripheral (Orlovska-Waast et al., 2019) overproduction of pro-inflammatory cytokines including interleukin-6 (IL-6), tumour necrosis factor-alpha (TNF-*α*), and C-reactive protein (CRP), which detrimentally affect neuroplasticity, stress hormone regulation, neuro-transmission, brain structure and function (Poletti et al., 2024; Wohleb et al., 2016; Khandaker et al., 2015), alongside abnormal levels of anti-inflammatory cytokines like IL-10, and growth factors such as transforming growth factor-*β* (TGF-*β*) (Moticka, 2016).

In depression, approximately one-third of individuals exhibit CRP levels compatible with low-grade inflammation (CRP>3 mg/L) (Osimo et al., 2020; Milaneschi et al., 2021), with these individuals being particularly prone to not responding to first- and second-line antidepressants (Strawbridge et al., 2015, 2019; Haroon et al., 2018; Vreijling et al., 2024; Arteaga-Henríquez et al., 2019). Similar patterns occur in SZ and BD, with elevated pro-inflammatory markers during acute episodes and potentially persisting during remission (Miller et al., 2011; Upthegrove et al., 2014; Mondelli et al., 2015; Modabbernia et al., 2013; Rosenblat and McIntyre, 2016). Across SMI, inflammatory changes manifest at both trait and state levels. Trait-level markers such as CRP represent persistent immune dysregulation, while state-dependent markers are associated with acute symptomatic states, particularly during manic and psychotic episodes (Skorobogatov et al., 2024). Immune dysregulation in SMI is also supported by alterations in immune cell phenotyping, including changes in lymphocyte and monocyte populations, with derived ratios serving as useful markers of systemic inflammation (Garcia and Cotner, 2025), as well as by abnormal regulation of immune-related genes, notably involving inflammatory pathways and major histocompatibility complex–associated loci (Ebrahimi et al., 2024). Importantly, considerable overlap exists in immune abnormalities across SMI diagnoses, indicating shared pathophysiological pathways and supporting transdiagnostic approaches (Morrens et al., 2022). These findings suggest the existence of immune-mediated subtypes across SMIs who may benefit from adjunctive anti-inflammatory treatments (Jha et al., 2025; Wessa et al., 2026a; Miller et al., 2025; De Picker et al., 2020; Cevoli et al., 2025).

The implication of inflammation in the pathophysiology of psychiatric disorders underscores its promise as a potential biomarker. However, the high dimensionality and complex interdependencies among immune, inflammatory, and neurotrophic markers pose substantial challenges to traditional analytical approaches. Furthermore, immune measures are inherently susceptible to biological and pre-analytical sources of variability, including fasting status, circadian rhythms, and measurement batch effects, which can substantially affect marker levels and compromise the reproducibility and long-term stability. Machine learning (ML) methods have gained increasing attention due to their ability to model non-linear relationships, integrate heterogeneous biological features, and predict outcomes in unseen observations (Bzdok and Meyer-Lindenberg, 2018; Fernandes et al., 2017).

Beyond their predictive utility, ML approaches may also play a critical methodological role in evaluating whether inflammatory and immune measures can meet key criteria for biomarker definition in SMI. Specifically, by enabling outcome-oriented modelling, feature selection, and potential validation across independent samples, ML methods allow immune markers to be evaluated in relation to predefined clinical endpoints, assessing their robustness, and generalizability. This, in turn, facilitates the delineation of the potential role of these markers for a personalized medicine in diagnostic, prognostic, predictive, and monitoring applications, in accordance with the Food and Drug Administration, Biomarker, EndpointS, and other Tools (FDA BEST) framework (FDA-NIH Biomarker Working Group et al., 2016), which provides a standardized taxonomy for defining biomarker categories and their intended clinical and regulatory use. We considered studies that employed both supervised and unsupervised ML approaches to analyse immune biomarkers, encompassing predictive, classificatory, and exploratory modelling frameworks. Supervised methods such as Random Forest (RF), Support Vector Machines (SVM), and k-Nearest Neighbours have been applied for differential diagnosis (Jahrami et al., 2024), or prediction of clinical outcomes such as treatment response and symptom severity (Chekroud et al., 2021; Jankowsky et al., 2024). Unsupervised approaches, including k-Means and Hierarchical Clustering, can be used to stratify patients and identify biological subtypes in both MDD (Kung et al., 2022; Lynall et al., 2020) and SZ (Gonul et al., 2025) (Table 1).

**Table 1:** Machine Learning principle and definitions.

Notion	Purpose
Supervised learning	Learn a mapping between input variables and a predefined outcome using labelled data, enabling prediction or classification of new observations.
Unsupervised learning	Identify underlying structure, patterns, or groupings in data without predefined outcome labels.
Feature or predictor	An input variable used by a machine learning model to explain, predict, or classify an outcome of interest.
Outcome or response	The target variable that a supervised learning model aims to predict or classify.
Regression	Model and predict a continuous outcome variable based on one or more input features.
Classification	Assign observations to discrete outcome categories based on learned patterns in the input data.
Clustering	Group observations into homogeneous clusters based on similarity across features without predefined labels.
Cross-validation	Iteratively training and testing the model on different data subsets (folds).
Training data	Subset of data used to fit model parameters and learn relationships between features and outcomes.
Test data	Independent dataset used to evaluate final model performance after training is complete.
Validation set	Dataset used during model development to tune parameters and select models without influencing final performance estimates.
Hyperparameter tuning	Optimise model configuration settings that are not learned directly from data, to improve performance and generalisation.
Feature selection	Identify and retain the most informative predictors while removing redundant or irrelevant features.
Dimensionality reduction	Reduce the number of input variables by transforming data into a lower dimensional representation while preserving relevant information.
Class imbalance	A condition in which outcome categories are unequally represented, potentially biasing model learning and performance.
Overfitting	When a model captures noise of the training data, resulting in poor generalisation to new data.
Bias	Systematic error introduced by data, model design, or assumptions that leads to consistently skewed predictions or conclusions.
Data leakage	When a ML model is trained using data from the test set, leading to inflated and misleading performance metrics.
AUC	Area Under the Curve, is a single scalar value that measures the overall performance of a binary classifier. he AUC value is within the range [0.5–1.0], where the minimum value represents the performance of a random classifier and the maximum value would correspond to a perfect classifier.
Accuracy	Is a metric that measures how often a machine learning model correctly predicts the outcome. Values ranges from 1 (correctly classifying every single instance) to 0 (zero correct prediction).
Balanced Accuracy	Is the average of sensitivity and specificity; accounts for class imbalance.

Previous reviews have examined ML applications in psychiatry across various domains (Dwyer et al., 2018; Bracher-Smith et al., 2021; Colombo et al., 2022; Lin et al., 2020), with immune-inflammatory markers achieving diagnostic accuracy rates up to 80% for differential diagnosis and over 85% for treatment response prediction (Colombo et al., 2022; Monopoli et al., 2025), though performance varies considerably depending on the specific markers included and sample characteristics (Osimo et al., 2020). However, to the best of our knowledge, no previous work has systematically examined performances or the methodological implementation of ML algorithms applied on immune biomarkers within SMI, nor comprehensively evaluated their different applications as described in the FDA BEST framework (Fda best glossary, 2025). In our context, we focused on biomarkers that play a role in immune system and inflammation, (e.g., cytokines, acute-phase proteins, growth factors and blood cells) which, for the scope of our review, can be employed in four BEST categories:

(1) *diagnostic* markers, to support diagnosis of SMI with respect to healthy controls (HC), other psychiatric disorders, or identification of subtypes within SMI populations, (2) *predictive* markers of treatment response, (3) *monitoring* to assess the clinical status of a disorder, and (4) *prognostic* markers for disease trajectory outcomes (e.g. remission). Thus, this systematic review aims to i) synthesize the evidence for ML algorithms applied to immune biomarkers in SMI; ii) evaluate their performance across different biomarker applications, using key metrics for supervised and unsupervised algorithms; and iii) critically assessing their methodological quality, with particular attention to immunology-specific sources of variability (including fasting status, timing of blood collection, and batch effects), as well as feature selection, outcome definition, and validation strategies, in order to appraise their potential for translation into clinically actionable tools.

## 2 Material and methods

### 2.1 Search strategy

We performed a PRISMA-compliant systematic search across four electronic databases: PubMed, Web of Science, Scopus and PsycINFO. The search covered all studies published up to 14 July 2025. The complete search strings, including all keywords, Boolean operators, and database-specific syntax, are available in the preregistered PROSPERO protocol (ID: CRD420251178464) and in the Supplementary Material (Material S1).

### 2.2 Eligibility criteria

Studies were considered eligible if they met the following criteria. Only human studies were included, focusing on individuals with SMI, namely major depressive (MDD), bipolar (BD) and schizophrenic spectrum disorders (SZ), as the primary diagnosis according to the Diagnostic and Statistical Manual of Mental Disorders (DSM) or the International Classification of Diseases (ICD). Eligible studies were required to apply ML-based computational approaches to study outcomes of interest (i.e., diagnosis, prognosis, monitoring and prediction) in SMI. In addition, studies had to include at least one of the following immune biomarkers as input features for ML models: cytokines, chemokines, interleukins, acute-phase proteins, immune cell phenotyping, white and red blood cell counts, platelets, growth factors, complement components, hemogram-derived ratios, as well as immune-related genes, RNA transcripts, or proteins. Only peer-reviewed articles were considered, including observational cohort studies, register-based studies, cross-sectional and longitudinal designs, as well as randomised controlled trials. Studies were excluded if immune biomarkers were not used as input features for ML models. Studies implementing systems biology approaches or relying exclusively on classical univariate statistical analyses, such as linear or logistic regression and mediation analyses were also excluded. Additionally, studies focusing on prenatal or perinatal populations were excluded due to the specific immune changes that occur during pregnancy, representing an unique biological context (Ding et al., 2022; Munoz-Suano A and Hamilton, 2011). Human studies in which SMI was not the primary diagnosis were not considered eligible. Finally, literature reviews, case reports, conference proceedings or abstracts, preprints, book chapters, meta-analyses, study protocols, and studies not written in English or Italian were excluded.

### 2.3 Screening

Duplicates were removed prior to title and abstract screening on Covidence (2025) by two authors (RS and FC). Full text screening was performed by three authors (RS, FC and PE), with each study assessed by two reviewers to ensure reliability. Data extraction was conducted independently by three authors (RS, FC, and PE) using a standardized extraction form. Disagreements at any stage were resolved between the authors.

### 2.4 Data extraction and data synthesis

For each study, we systematically extracted detailed information on the study population including sex, age, diagnostic group with sample size, and the presence of chronic inflammatory or immune-related comorbidities. Input data for ML algorithms characteristics were also recorded, encompassing biological sample type, laboratory assays, handling of missing data, multimodal data integration, and adjustment for relevant covariates. Pre-analytical variables specifically relevant to immune markers were also systematically recorded, including fasting status, timing and conditions of blood collection, processing and storage procedures, and the reporting of batch-effect correction strategies. We then extracted information on the ML approach employed, including specific algorithms used and distinguishing between supervised and unsupervised methods, information on validation procedures, including internal validation, hold-out test sets, and external validation on independent samples, together with class imbalance, feature selection strategies and model interpretability. In order to evaluate ML algorithm performances, we extracted appropriate metrics for each ML model. For supervised classification models, extracted metrics included the area under the receiver operating characteristic curve (ROC-AUC), accuracy, and balanced accuracy. When balanced accuracy was not explicitly reported, it was calculated from sensitivity and specificity as follows:

BalancedAccuracy=(Sensitivity+Specificity)/2

For supervised regression models, performance was assessed using the coefficient of determination (R2) and the root mean square error (RMSE). For unsupervised clustering approaches, internal validation metrics such as the silhouette score were recorded when available. In total, 48 variables were extracted (Supplementary Table S1). We then present a systematic data synthesis, resuming data for SMI category, biomarker type and origin, ML task, and intended biomarker application according to FDA BEST framework (diagnostic, predictive, monitoring or prognostic). When available, we included the algorithms’ performance metrics, (specifically the AUC as it is the most common metric), and, if not available, accuracy or balanced accuracy. For descriptive purposes, not to be considered pooled estimates, performance metrics were summarized using ranges (minimum–maximum), other descriptive statistics were reported in mean and standard deviations. Analyses were performed using R (version 4.5.0) (R, 2025). R Markdown code used for data extraction and synthesis is available from the authors upon request.

## 3 Results

**Fig. 1:**
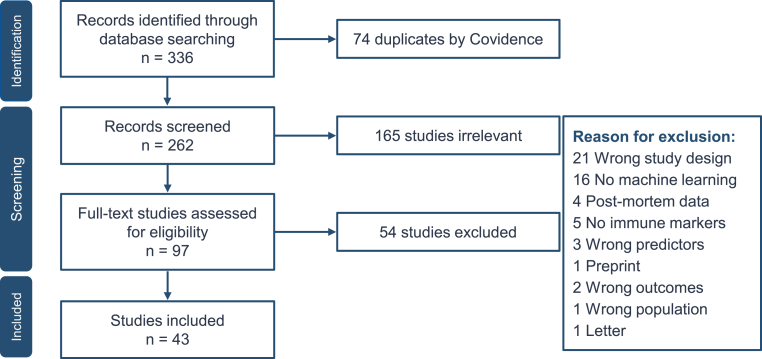
PRISMA flowchart of the review process and studies selection.

After removing duplicates, 262 studies advanced to the screening phase, as shown in the PRISMA flow chart (Fig. 1). Of these, 43 met the inclusion criteria (Table 2) with a total sample of 11,556 participants, 8339 with SMI (4410 females, 52.88%) and 3217 healthy controls (HC) (1552 females, 48.24%; 449 missing information), with average age of 36.95 (*±* 10.12). Most of the studies used a cross-sectional retrospective design (n = 24), followed by cross-sectional prospective (n = 11), prospective randomized controlled trials (n = 4), longitudinal retrospective (n = 3), and longitudinal prospective (n = 1). Heterogeneity in mental illness diagnosis across studies was handled by grouping into three main diagnostic categories: MDD, BD, SZ. MDD was assessed in 22 studies involving the wider sample of 3228 participants. BD were investigated in 17 studies, including Bipolar II disorder (n = 399), Bipolar I disorder (n = 295), and for generally identified as affective disorder (i.e., unspecified mood disorders or studies without distinction between specific diagnoses) (n = 73) and bipolar disorder not otherwise specified (n = 1847). SZ were represented in 22 studies, comprising schizophrenia (n = 1637), schizoaffective disorder (n = 328), schizophreniform disorder (n = 31), and psychotic disorder not otherwise specified (n = 501). One study (Popov et al., 2024), including 90 SZ patients, omitted the exact participant numbers for diagnostic subgroups. Regarding chronic inflammatory or immune-related comorbidities, 25 studies excluded individuals with these disorders, 5 included them, and 13 did not specify.

**Table 2:** Review of included studies.

Study author (s) year	Sample size and diagnosis	Immune biomarkers	ML algorithms	Algorithm objective	Best algorithm performance	Most relevant predictors
Kozyrev EA. et al. (2023)	SZ = 217, HC = 90	IL-1α, IL-1β, IL-1, IL-1RA, IL-2, IL-3, IL-4, IL-5, IL-6, IL-7, IL-8, IL-9, IL-10, IL-12P40, IL-12P70, IL-13, IL-15, IL-17A, TNF-α, TNF-β, Flt-3L, IFN-α2, IFNγ, CCL2, CCL3, CCL4, CCL7, CXCL1, CXCL10, CCL11, CCL22, EGF, tgf-α, PDGF-AA, PDGF-AB/BB, FGF-2, G-CSF, GM-CSF	DT, KNN, SVM, ANN	diagnosis	DNN: SZ vs HC BA 0,695∗	CCL22
Lizano et al. (2021)	SZ = 50, SZA = 29, BD1 n = 61, HC = 60	IL-1β, IL-6, IL-8, IL-10, IL12/IL23p40, IFNγ, TNF-α, TNF-β, CRP, Flt-1, VEGF, VEGFC, VEGFD, TGFβ1, C4a	PCA, HCL	subtyping	Silhouette score = 0.59	CRP, IFNγ, IL-1β, IL-8, IL-10, TNF-α, VEGF
Shaojia Lu et al. (2013)	CTE (TD) = 22, CTE (NTD) = 21, HC = 22	AgRP, b-FGF, BTC, GITR-L, CXCL11, IL-1β, IL-1 R1, CCL28, NT-4, TGF-β3, CCL25, TRAIL-R4, VEGF	HCL	subtyping	NA	AgRP, b-FGF, BTC, GITR-L, I-TAC, IL-1β, IL-1 R1, MEC, NT-4, TECK, TGF-β3, TRAIL-R4, VEGF
Popov P. et al. (2024)	SZ = 90, HC = 40	immune-inflammatory responses system, M1 macrophage, T helper-17-axis	LDA, SVM	subtyping, diagnosis	SVM: MNP vs HC accuracy = 0.978	T helper-17 axis, M1 macrophage activation, M1 macrophage profILe
Shen He et al. (2019)	MDD = 40	IL-1β, IL-6, CRP, Beclin-1	RF	response	RF: responder vs non-responder AUC = 0.815, acc = 0.791	beclin-1
Lee Yena et al. (2021)	BD = 60, HC = 30	TNF-α, sTNFR1, IL-1β, IL-2, IL-4, IL-6, IL-8, IL-10, IL-12	DT, KNN	response	DT: R2 = 0.22, RMSE = 0.08 in predicting reductions in anhedonic symptoms	IL-4, IL-6, IL-8, IL-1β, IL-2, IL-12, sTNFR1, IL-10, TNF-α, p-NFκB, p-FADD, p-IKKα/β, TNFR1, p-IRS1, p-p38, p-JNK
Lusi Zhang et al. (2023)	SZ = 41, SZA = 27, BD1 = 61, HC = 55	CRP, IL-6, C4a, IFNγ, IL-8, IL-10, VEGFD	HCL, PCA	subtyping	Silhouette score = 0.55	CRP, C4a
Elbakary et al. (2025)	MDD = 94	WBC, RBC, Hgb, Hct, MCV, RDW, Platelet, MPV, ANC, Lymphocyte, Monocyte, EosinophIL	RF	response	RF: responder vs non-responder BA = 0.623, AUC = 0.59	PLR
Benedetti et al. (2021)	MDD = 108	IL−1β, IL-1rα, IL-2, IL-4, IL-5, IL-6, IL-7, IL-8, IL-9, IL-10, IL-12(p70), IL-13, IL-15, IL-16, IL-17, IFNγ, TNF-α, MCP1/CCL2, MIP-1α/CCL3, MIP-1β/CCL4, RANTES/CCL5, CCL11, IP-10/CXCL10, FGF, G-CSF, GM-CSF, PDGF-B, VEGF	Elastic Net	response	Elastic Net: BA = 0.63, AUC = 0.75	IL-1β, IL-12, TNF-α
Popescu et al. (2025)	SZ = 70	CRP, ESR	RF, SVM	severity	NA	CRP
Al-Hakeim et al. (2019)	MDD = 60, HC = 30	CRP, IL-6, IL-10	SVM, LDA, PCA	diagnosis	SVM: MDD vs HC accuracy = 100% in validation samples LDA: MDD vs HC accuracy = 0.978 in validation samples	IL-10, IL-6
Guilloux et al. (2015)	MDD = 34, HC = 33	CD3D, CD97, IFITM3, GZMA, TAGLN2, TIMP1, PSMA4, PSMA6	SVM	response	SVM: 13-gene model: accuracy = 0.794 accuracy, BA = 0.781∗	IFITM3, TIMP1
Dargél et al. (2018)	BD = 635	CRP	RF	trajectory, diagnosis	accuracy = 0.849, BA = 0.847	CRP
Martinuzzi et al. (2019)	PSY = 325	IL-6, IL-7, IL-8, IL-10, IL-12p40, IL-15, IL-16, IL-17, IL-18, IL-21, IL-23, IL-27, IFNγ, ccl2, CCL3, CCL4, CCL11, CCL13, CCL17, CCL19, CCL20, CCL22, CCL26, CCL27, CX3CL-1, CXCL10, CXCL11, CXCL12, TNF-α, TNF-β, VEGF, CRP, SAA, sICAM-1,sVCAM-1	Elastic Net, K-sparse	subtyping, response	AUC = 0.73, BA = 0.64∗	IL-15, CXCL12
Göteson et al. (2022)	BD = 324, HC = 169	ADA, AGER, MCP-4, CCL19, MCP-1, CCL20, CCL23, CCL25, CCL28, CCL3, CCL4, CCL7, CCL8, CXCL10, CXCL11, CXCL13, CXCL16, CXCL5, CXCL6, CXCL9, CD40-L, CHI3L1, CDCP1, SELE, CD69, ESM-1, ECP, CCL11, Flt3L, CX3CL1, NA, CXCL1, HAVcr-1, ICOSLG, IFNγ, IL-1 alpha, IL-1rl1, IL-1ra, IL-10, IL-10RA, IL-10RB, IL-12, IL-12B, IL-13, IL-15RA, IL-17RB, IL-17A, IL-17C, IL-18, IL-18R1, IL-2, IL-2RB, IL-20, IL-20RA, IL-22RA1, IL-24, IL27-A, IL33, IL-4, IL-5, IL-6, IL-6RA, IL-7, IL-8, IL-16, MMP-1, LAP tgfβ1, TNF, TNF-β, LIF, LIF-R, ILT-3, LITAF, CSF-1, MMP-7, MIC-A, MYD88, MPO, CD244, OSM, LOX-1, PSGL-1, PTX3, PECAM-1, PD-L1, S100A12, SLAMF1, CD6, CD5, TR-AP, TSLP, TRAIL, TRANCE, TWEAK, BAFF, TNFSF14, FASLG, TRAIL-R2, OPG, TNF-R1, TNF-R2, TNFRSF4, CD40, FAS, TNFRSF9, LYN, PTPN22, uPA, uPAR, VEGF-A, VEGF-D, VEGFR-2	RF	diagnosis	accuracy = 0.56, AUC = 0.72	TGF-α, EGF, MMP-7, SRC, GDF-15, CCL4
Wollenhaupt-Aguiar et a. (2020)	BD2 = 54, MDD = 54, HC = 54	IL-2, IL-4, IL-6, IL-10, TNF-α, IFNγ, IL-17A	SVM	diagnosis	MDD vs HC: AUC = 0.74, BA = 0.69∗	IL-6, carbonyl, BDNF, IL-10, IL-17A, IL-4, TNF-α
Zeng J et al. (2023)	MDD = 918, BD2 = 242	WBC, monocyte, monocyte ratio, neutrophil, neutrophil ratio, Basophils, Basophils ratio, eosinophil, eosinophil ratio, lymphocyte, lymphocyte ratio, RBC, HCT, MCV, RDW-CV, HGB, MCH, MCHC, RDW-SD, PLT, PDW, MPV, P-LCR, NLR, PLR, MLR	SVM, RF, XgBoost	diagnosis	XgBoost BD2 vs MDD: BA = 0.835∗, accuracy = 0.863, AUC = 0.889	NA
Poletti et al. (2021)	BD = 81, MDD = 127, HC = 32	IL-1β, IL-1rα, IL-2, IL-4, IL-6, IL-7, IL-8, IL-9, IL-10, IL-13, IL-16, IL-17, IFNγ, TNF-α, MIF, CCL1, CCL2, CCL3, CCL4, CCL5, CCL8, CCL7, CCL11, CCL13, CCL15, CCL17, CCL19, CCL20, CCL21, CCL22, CCL23, CCL24, CCL25, CCL26, ccl27, CXCL1, CXCL2, CXCL5, CXCL6, CXCL8, CXCL9, CXCL10, CXCL11, CXCL12, CXCL13, CXCL16, CX3CL1, bFGF, PDGF-B, VEGF	Elastic Net	diagnosis	Elastic Net BD2 vs HC: BA = 0.94, AUC = 1	CCL3, CCL4, CCL5, CCL11, CCL25, CCL27, CXCL6, CXCL11, IL-9, TNF-α
Érico de Moura SILveira J et al. (2019)	MDD = 43, BD = 61, SZ = 55, AN = 41	IL-6, IL-1β, IL-10, TNF-α, CCL11	SVM, RF, ANN	trajectory	AUC = 0.83, BA = 0.729∗	NA
Donnelly et al. (2025)	MDD = 309	CRP, IFNγ, IL-1α, IL-2, IL-2RB, IL-4, IL-5, IL-6, IL-7, IL-8, IL-10, IL-10RA, IL-10RB, IL-12B, IL-13, IL-15RA, IL-17A, IL-17C, IL-18, IL-18R1, IL-20, IL-20RA, IL-22RA1, IL-24, IL-33, TG-Fα/TNF, TNF-β, TNFRSF9, TNFSF14, TRAIL, TRANCE (RANKL), thymic stromal lymphopoietin (TSLP), TWEAK, CCL3, CCL4, CCL11, CCL19, CCL20, CCL23, CCL25, CCL28, CX3CL1, CXCL1, CXCL5, CXCL6, CXCL9, CXCL10, CXCL11, CCL2, CCL8, CCL7, CCL13, CD244, CD40, CD5, CD6, CD8A, CDCP1, FGF5, FGF19, FGF21, FGF23, HGF, VEGFA, FLT3L, CSF1, SCF, basophILs, eosinophILs, lymphocyte, monocyte, neutrophIL, platelet, RBC, WBC, Hb, Hct, MCH, MCHC, MCV leukemia inhibitory factor (LIF), LIFR, oncostatin M (OSM), SLAMF1, PD-L1	Elastic Net	subtyping	MDD diagnosis: BA = 0.580	Immuno metabolic set
SG FILlman et al. (2015)	SZ = 43, HC = 42	IL-1β, IL-2, IL-6, IL-8, IL-18	SPSS TwoStep Clustering	subtyping	NA	IL-6, IL-8, TNF-α
Dung Hoang et al. (2022)	FES = 47, HC = 33	IL-1β, IL-6, IL-8, IL-10, IL12/IL23p40, IFNγ, TNF-α, sFlt-1, bFGF, PlGF, VEGF	PCA, HCL	subtyping	Silhouette score = 0.46	IL-1β, IL-6, IL-8, TNF-α
Ucuz et al. (2020)	SZ = 57, BD = 23, MDD = 61, AN = 24, HC = 70	leukocyte, lymphocyte, neutrophIL, monocyte, platelet, NLR, PLR, MLR, MPV	ANN	diagnosis	Accuracy = 0. 99	leukocytes, lymphocyte, monocyte, neutrophil, MPV, NLR, MLR
He S et al. (2024)	MDD = 83, HC = 50	CD163, Syndecan-1, IL-1R2, IL-11, CXCL14, VEGFR1, GITR, XEDAR, CRTAM, RANK, Trem1, HLADR, B cells, CD36, Th17, Treg	Elastic Net, Lasso, Ridge, GBM, XgBoost, Superm PC, SVM, RF	trajectory, response	RF trajectory responder vs non-responder: accuracy = 0.895, AUC = 0.944, Ridge diagnosis MDD vs. HC Ridge accuracy = 0.920, AUC = 0.864	NA
Tao YL et al. (2025)	HC = 100, MD = 41, SD = 31	IL-17	ANN	severity	discovery cohort: HC AUC = 0.95, mILd MDD AUC = 0.87, severe MDD AUC = 0.87	IL-17
Kittel-Schneider et al. (2020)	BD = 70, MDD = 42	IL-18, MDC, MIP-1 beta, MIF, ANG-2, MMP-1, MMP-3, MMP-7, MMP-9, MMP-10, MCP-1, MCP-2, MCP-4, MIG, MPIF-1, MPO, B2M, Osteopontin, CRP, CD40, PAI-1, CD40-L, CD5L, HCC-4, PARC, RAGE, C3, Resistin, E-Selectin, EN-RAGE, SCF, Eotaxin-1, RANTES, ENA-78, TN-C, FAS, TIMP-1, TRAIL-R3, IgA, IgM, TNFR2, VCAM-1, ICAM-1, VEGF, IP-10, IL-6r, IL-8, IL-16, AXL, SAP, CLU, HGF, SCF, NGAL, SortILin, Vitronectin, THP, PLGF, SOD-1	AdaBoost	diagnosis	BA = 0.67	PDGF-BB, TSP-1
Liu C et al. (2025)	BD = 28, MDD = 40 52 finished rTMS	CRP, TNF-α	Extra Trees, RF, GBM, XgBoost	diagnosis	GBM: AUC = 0.863, accuracy = 0.773	CRP
Yee JY et al. (2025)	SZ = 146, HC = 49	4E-BP1, ADA, AXIN1, CASP-8, CCL11, CCL19, CCL20, CCL23, CCL25, CCL28, CCL3, CCL4, CD244, CD40, CD5, CD6, CD8A, CDCP1, CSF-1, CST5, CX3CL1, CXCL1, CXCL10, CXCL11, CXCL5, CXCL6, CXCL9, DNER, EN-RAGE, FGF-19, FGF-21, FGF-23, FGF-5, Flt3L, GDNF, HGF, IFNγ, IL-10, IL-10RA, IL-10RB, IL-12B, IL-15RA, IL-17A, IL-17C, IL-18, IL-18R1, IL-6, IL-7, IL-8, tgfβ1, LIF-R, MCP-1, MCP-2, MCP-3, MCP-4, MMP-1, MMP-10, NT-3, OPG, OSM, PD-L1, SCF, SIRT2, SLAMF1, STAMBP, TGF-α, TNF-α, TNF-β, TNFRSF9, TNFSF14, TRAIL, TRANCE, TWEAK, uPA, VEGFA	SVM	subtyping	Antipsychotic Response (ARE vs. CRE & CRT) BA = 0.780, AUC = 0.884	CCL25, CST5, CD5, TNFRSF9, MMP10
LS Sæther et al. (2022)	SZ = 175, SZA = 43, schizophreniform = 31, NOS = 94, BDI = 173, BDII = 103, BDNOS = 14, HC = 770	Inflammatory/Immune Canonical Variate	HCL	subtyping	Silhouette score = 0.37	IL-18 system cytokines, BD-2, VCAM-1
Sun L et al. (2024)	MDD = 20, HC = 20	TRPV2, ZNF713, CTSL	RF, Lasso, SVM-RFE	diagnosis	BA = 0.867, accuracy = 0.764	CTSL, PSME2, ZNF713, CXCL8, TRPV2
Domenici et al. (2010)	MDD = 245, SZA = 229, HC = 254	RANTES, TIMP-1, EGF, MMP-9, TNFRII	RF, LDA	diagnosis	NA	MDD: insulin, MMP-9. SZ: BDNF, RANTES, EGF, TIMP-1
Lalousis et al. (2023)	SZ = 467, HC = 600	CRP, IFNγ, IL-10, IL-12, IL1-β, IL-2, IL-6, IL-8, TNF-α	HYDRA	subtyping	Adjusted Rand Index = 0.573	IL-6, IL-8, CRP, IL-10
Luo X et al. (2025)	SZ = 64, MDD = 53, HC = 33	CCL11, CCL20, EGF, G-CSF, GM-CSF, IL-15, PDGFAA, CCL5, VEGF	RF, XgBoost	diagnosis	XgBoost MDD vs HC: AUC = 0.994, accuracy = 0.885, BA = 0.869∗	NA
Martinuzzi et al. (2021)	MDD = 264, BD = 198	CCL2, CCL3, CCL4, CCL11, CCL13, CCL17, CCL22, CXCL10, IL-6, IL-7, IL-8, IL-10, IL-12/IL-23 p40, IL-15, IL-16, IL-27, IFNγ, TNF-α	Elastic Net	diagnosis	Discovery cohort: accuracy = 0.83, AUC = 0.83	IL-10, IL-15, IL-27
Foiselle et al. (2022)	SZ = 238, SZA = 72	IL-6, IL-7, IL-8, IL-12/IL-23p40, IL-15, IL-16, IL-17A, TNF-α	Elastic Net	subtyping	NA	IL-6, IL-7, IL-12/23 p40, IL16, TNF-α
Skorobogatov et al. (2024)	BD = 321, SZ = 186, 75 HC = 185	IL-6, IL-8, IL-12/IL-23p40, IL-17, TNF-α, IFNγ, TRP, KYN, KA, QUINA, XA, QUINO, PICO	bootstrap forests	diagnosis, staging	Diagnosis SZ vs HC: AUC = 0.897, accuracy = 0.715	IL-6, TNF-α
Besga et al. (2015)	LOBD = 32, AD = 37, HC = 26	IL-1, IL-6, TNF-α	RF, DT, Naive Bayes	diagnosis	SVM LOBD vs HC: AUC = 0.9817, accuracy = 0.972	IL-6, TNF-α
Khoodoruth et al. (2025)	SZ = 69, HC = 30	NLR, PLR, MLR, CRP	RF	subtyping, response	TRS vs. NTRS + PANSS: accuracy = 0.884, AUC = 0.895	NA
Sánchez-Carro et al. (2023)	MDD = 91, HC = 80	TNF-α, CRP	RF, SVM, GBM	diagnosis, subtyping	melancholic vs. non-melancholic: AUC = 0.76	TNF-α, CRP
Liu X et al. (2022)	Affective disorders = 73, psychotic disorders n = 35	IL-8	DT	diagnosis	Accuracy = 0.861, AUC = 0.9	DBI, APOA1, LDH, ADL, CREA, MON, IL-8
Zhang Q et al. (2021)	SZ = 37, HC = 35	interaction terms	DT, RF, SVM	diagnosis	SVM accuracy = 0.986, AUC = 0.971	Cortisol × TNF-α × IL-8
Martínez-Cao et al. (2024)	SZ = 212	PLR, NLR, MLR	genetic algorithms, SVM-RFE	staging	Overall accuracy = 0.62, BA = 0.698∗, AUC = 0.503	PLR, NLR, MLR, IL-6
Malashenkova et al. (2023)	SZ = 63, HC = 36	TNF-α, IFNγ, CRP, cortisol, CIC, IL-1β, IL-8, IL-1RA, IL-4, IL-10, IgA, IgM, IgG	QDA, linear SVM, RBF SVM, KNN, Gaussian processes, naive Bayes classifier, DT, AdaBoost, RF, XgBoost	diagnosis	AdaBoost SZ vs HC: AUC = 0.71, accuracy = 0.78	IL-8, CIC, IL-4, IFN-γ, TNF-α, cortisol

Abbreviations: AD, Alzheimer Disease; AN, Anxiety disorder; ANN, Artificial Neural Network; ARE, antipsychotics-responsive; BD, bipolar disorders; BD1, bipolar disorder type 1; BD2, bipolar disorder type 2; BDNF, Brain-derived neurotrophic factor; BDNOS, bipolar disorder not otherwise specified; CCL, chemokine (C-C motif); CRE, clozapine-responsive; CRP, C-reactive protein; CRT, clozapine-resistant; CTE (NTD), childhood trauma exposure (non depression); CTE (TD) childhood trauma exposure (depression); CXCL, chemokine (C-X-C motif); DT, Decision Tree; EGF, epidermal growth factor; FES, first episode schizophrenia; FGF, fibroblast growth factor; GBM, Gradient Boosting; GDNF, glial cell line-derived neurotrophic factor; HC, healthy controls; HCL, Hierarchical Clustering; HGF, Hepatocyte Growth Factor; ICAM-1, Intercellular Adhesion Molecule 1; IFN, interferon; Ig, immunoglobulin; IL, interleukin; KA, kynurenic acid; KNN, k-nearest neighbours; KYN, kynurenine; LDA, Linear Discriminant Analysis; LOBD, late-onset bipolar disorder; ML, machine learning; MLR, monocyte-lymphocyte ratio; MDD, Major Depressive Disorder; NLR, neutrophil-lymphocyte ratio; NOS, psychosis not otherwise specified; NTRS, non treatment resistant; PCA, Principal Component Analysis; PICO, picolinic acid; PLR, platelet-lymphocyte ration; PSY, psychosis; QDA, Quadratic Discriminant Analysis; QUINA, quinaldic acid; QUINO, quinolinic acid; RBF, radial basis function kernel; RF, Random Forest; RFE, Recursive Feature Elimination; rTMS, repetitive transcranial magnetic stimulation; SVM, Support Vector Machine; SZ, schizophrenia disorders; SZA, schizoaffective disorder; TNF, tumor necrosis factor; TRP, tryptophan; TRS, treatment resistant; VCAM, vascular cell adhesion molecule; VEGF, Vascular endothelial growth factor; XA, xanthurenic acid.

### 3.1 Overview of ML input data and algorithms

**Fig. 2:**
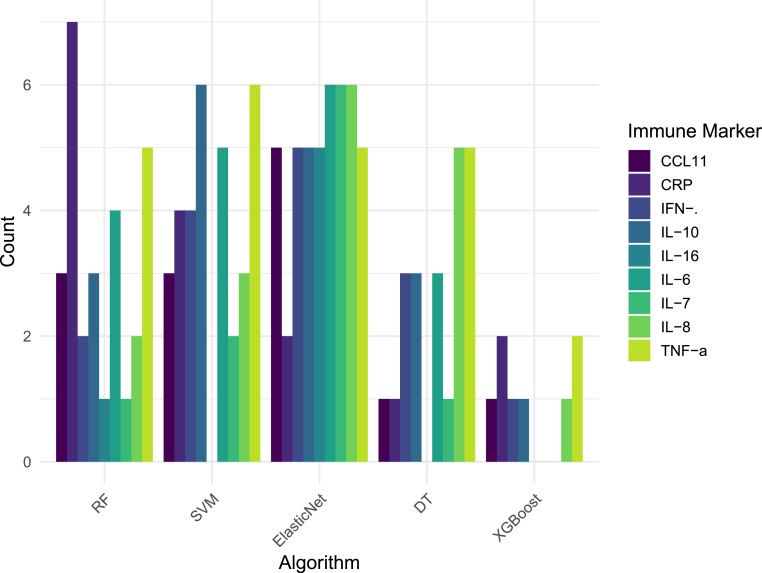
Multiple barplot shows the counts of how many times different immune markers are used as input features across the most used ML algorithms. Abbreviations: CCL, chemokine (C-C motif); CRP, C-reactive protein; DT, Decision Tree; IFN, interferon; IL, interleukin; RF, Random Forest; SVM, Support Vector Machine; TNF, tumour necrosis factor; XGBoost, Extreme Gradient Boosting.

In terms of ML input data, the most common immune biomarkers used as input features were IL-6 (n = 21), IL-8 (n = 19), TNF-*α* (n = 18), IL-10 (n = 17), IFN-*γ* (n = 15) and CRP (n = 14) (Fig. 2, Supplementary Fig. S2, see Tables S2,S3,S4 for more details stratified for diagnosis and algorithm). Most of the studies derived immune markers from serum (n = 21) and plasma (n = 10), with only two studies failing to report the sample origin. The most frequently employed platforms for analysing the samples were multiplex assays (n = 15), ELISA (n = 13), and immunoassays (n = 7). We also systematically extracted information on potential sources of pre-analytical heterogeneity in biomarker collection and processing. Fasting status was reported in only 20 of 43 studies (fasting, n = 18; non-fasting, n = 2; not reported, n = 23), sampling time in 19 (7–10 a.m., n = 17; variable, n = 2; not reported, n = 24), and blood processing and storage procedures in 22 (not reported, n = 15), with only 10 studies reporting the interval between collection and processing (0 min, n = 2; 15 min, n = 3; 30 min, n = 2; 1 h, n = 3; not reported, n = 33). Batch-effect correction was reported in only 6 studies (not reported, n = 37). Overall, the majority of studies failed to report one or more key pre-analytical variables and batch-effect correction, representing a critical gap in transparency that substantially limits the reproducibility and cross-study comparability of the reported immune markers. Another source of heterogeneity arises from data transformation during preprocessing. Logarithmic transformation was applied in 12 studies, standardization in 8, normalization in 5, and other methods in 6.

Across all studies, the mean number of collected immune biomarkers was 41.51 ± 81.42, though the average number of features actually used as model inputs was considerably lower (18.74 ± 29.09). Four studies considered a single immune marker, 21 included between 2 and 10, 9 between 11 and 20, and 9 more than 20 analytes, with 12 studies applying feature selection to reduce a broader initial panel. Missing data were most commonly managed by excluding analytes exceeding a predefined missingness threshold (e.g., >10%, >20%, or >30%; n = 10) or by imputing values at the lower limit of detection (n = 5). Only three studies employed methodologically rigorous imputation techniques, such as multivariate imputation by chained equations or Markov Chain Monte Carlo, while three others did not report the method used. Nineteen studies provided no information on missing data handling, and four did not specify the degree of missingness.

Regarding features integration, 29 studies adopted a multimodal approach, combining immune biomarkers with other biological data (n = 22), such as Brain-Derived Neurotrophic Factor (BDNF) and triglycerides, clinical and demographic variables (n = 16), including age, sex, and the Positive and Negative Syndrome Scale (PANSS), and cognitive data (n = 7); two studies incorporated all three modalities within the same model. Half of the studies (n = 22) accounted for potential confounders, either by including covariates directly in the model, regressing their effects out from immune biomarkers prior to modelling, or incorporating adjustment steps within the pipeline.

**Fig. 3:**
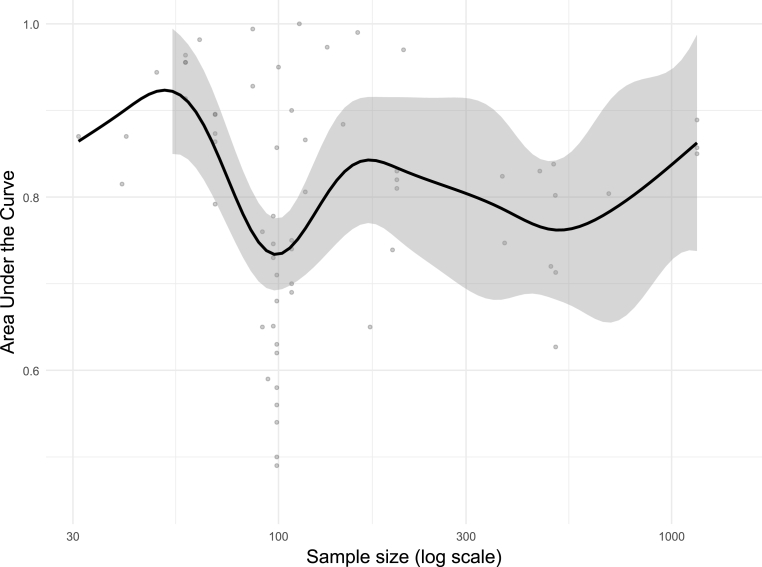
Relationship between model performance and sample size. Area Under the Curve as a function of sample size. Points represent individual model runs; black line shows GAM-smoothed trend with 95% confidence interval (grey shaded region). The x-axis is log10-scaled.

**Fig. 4:**
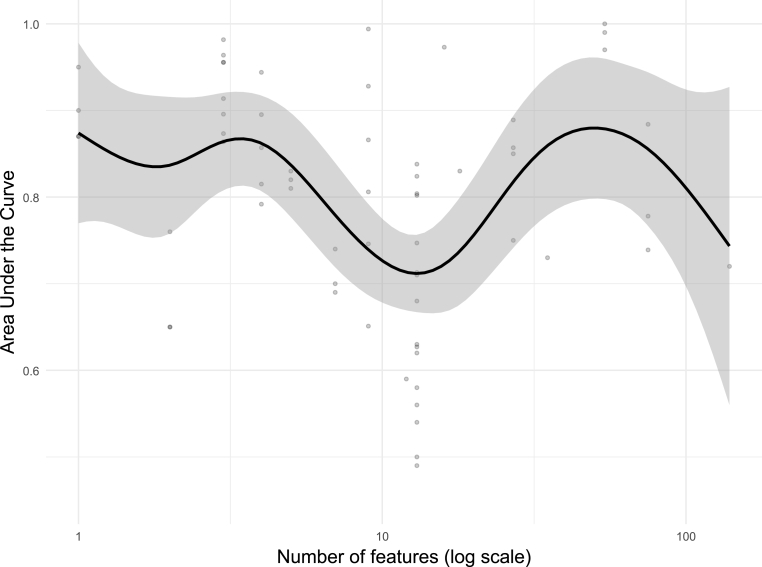
Relationship between model performance and sample size. AUC as a function of the number of input features (immune markers). Points represent individual model runs; black line shows GAM-smoothed trend with 95% confidence interval (grey shaded region). The x-axis is log10-scaled.

In terms of algorithms, the majority of studies employed supervised classification approaches (n = 36), with Random Forest (RF; n = 17), Support Vector Machine (SVM; n = 12), Elastic Net logistic regression (n = 7), Decision Trees (DT; n = 6), and XGBoost (n = 5) being the most commonly used. Three studies applied supervised regression algorithms, and seven adopted unsupervised learning, primarily to investigate SMI subtypes through Hierarchical Clustering (n = 6) or TwoStep Clustering (n = 1). With respect to model evaluation, cross-validation (CV) was employed in 33 studies, most commonly using 10-fold CV (n = 14), Leave-One-Out Cross-Validation (LOOCV; n = 7), or nested CV (n = 6), with hyperparameter tuning performed in 22 studies. Sixteen studies used a training-test split, with an 80/20 ratio being the most frequent (n = 6); however, 13 of these performed preprocessing steps prior to the split, introducing a risk of data leakage and potentially inflated performance estimates. External validation was performed in only six studies, and while class imbalance was present in 27 studies, only eight applied corrective measures such as class weighting techniques. Feature selection and interpretability methods were reported in 38 studies. The most common feature selection approaches were Recursive Feature Elimination (RFE; n = 7), feature importance ranking (n = 6), and LASSO (n = 4), while model interpretability was primarily assessed through feature importance scores (n = 20) and SHapley Additive exPlanations (SHAP; n = 5). Dimensionality reduction was applied in only 15 studies, mainly via Principal Component Analysis (n = 8) and penalized regression methods (n = 4). Notably, 12 studies had fewer than 10 samples per feature without applying dimensionality reduction, raising concerns about model overfitting. To examine factors influencing model performance, AUC values were plotted as a function of both input feature dimensionality and sample size (Fig. 3, Fig. 4). Higher AUC values were observed in studies using fewer than 10 input features and in datasets with fewer than 50 observations, likely reflecting performance overestimation due to overfitting rather than true discriminative ability. A modest decline in AUC was evident with moderate feature sets (10–50 features) and medium-sized samples (100–500 observations), potentially indicating more stable and realistic estimates. Performance increased again when more than 50 features or more than 500 observations were included, consistent with improved generalizability and statistical power.

### 3.2 Overview of results according to the FDA BEST framework

Individual model performance showed substantial variability, with AUC values ranging from 0.590 to 1.0; (Table 2). The performance metrics per each FDA BEST are summarized in Table 3. The full set of immune biomarkers, ML models and performances is available in Tables S5 and S6 and Supplementary Material 2. In the following sections, we reported the main findings divided for FDA BEST category, for each we included information regarding the input immune biomarkers and ML algorithms employed with the respective performances and sample size.

**Table 3:** ML algorithm performances.

Algorithm objective	SMI	AUC	n	Accuracy	n	BA	n
Diagnostic (SMI vs control)	MDD	0.650 – 0.990	9	0.764 – 1.000	6	0.690 – 0.890	8
	BD	0.700 – 1.000	12	0.560 – 0.972	5	0.515 – 0.940	6
	SZ	0.651 – 0.857	17	0.700 – 0.986	17	0.729 – 0.986	6
Diagnostic (differential)	MDD vs BD	0.690 – 0.970	6	0.822 – 0.863	4	0.670 – 0.900	7
	BD vs SZ	0.627	1	-	0	-	0
	SZ vs MDD	0.806 – 0.866	2	0.722 – 0.778	2	0.731 – 0.769	2
Diagnostic (subtyping)	MDD	0.76	1	–	0	–	0
	BD	–	0	0.99	1	–	0
	SZ	0.739	1	0.763 – 0.990	4	0.692	1
Predicting	MDD	0.590 – 0.944	5	0.659 – 0.895	4	0.623 – 0.630	2
	BD	–	0	–	0	–	0
	SZ	0.778 – 0.895	4	0.724 – 0.884	4	0.780 – 0.867	2
Monitoring	MDD	0.870 – 0.950	3	–	0	–	0
	BD	0.713 – 0.838	3	–	0	–	0
	SZ	0.713 – 0.838	3	0.62	1	0.698	1
Prognostic	MDD	–	0	–	0	0.479 – 0.580	3
	BD	–	0	–	0	–	0
	SZ	0.73	1	–	0	0.64	1

Per each SMI and algorithm objective report the ranges (min-max) of model performance: AUC (Area under the curve), accuracy and BA (Balanced Accuracy) reporting the number (n) of models with reported metrics. Abbreviations: BD, bipolar disorder; MDD major depressive disorder; SZ, schizophrenia.

### 3.3 Diagnostic markers

Diagnostic outcomes were evaluated across 73 models, which incorporated an average of 15.63 immune markers as input features (SD = ±21.1; range: 1–139). The most common analytical approaches for case-control classification were RF and SVM, with TNF-α, IL-10, CRP, and IL-6 representing the most frequently used immune biomarkers. Diagnostic performance was broadly comparable across disorders when patients were compared with healthy controls (HC): AUC values ranged from 0.650 to 0.990 in MDD (n = 9), 0.700 to 1.000 in BD (n = 12), and 0.651 to 0.857 in SZ (n = 17). It should be noted, however, that case-control classification relative to HC has limited real-world clinical relevance, as distinguishing patients from healthy individuals offers little actionable value in practice.

Differential diagnosis studies focused primarily on distinguishing MDD from BD (n = 6), achieving a range of AUCs 0.690–0.970. Performances ranged from modest (AUC 0.69, n = 162) with individual cytokines (Wollenhaupt-Aguiar et al., 2020), to high (AUC = 0.889, n = 1160) with complete blood count metrics (Zeng et al., 2023). The most accurate classification (AUC = 0.97, n = 240) was achieved when considering biomarker panels as input features. Specifically, Poletti and colleagues (Poletti et al., 2021) applied an elastic net penalized regression model in a prospective design, demonstrating that BD exhibits primarily a pro-inflammatory signature, while MDD is distinguished by a combined pro-inflammatory and regulatory profile. Differential diagnosis between MDD and SZ was retrospectively explored by Luo and colleagues (Luo et al., 2025), with several cytokines resulting significant (e.g. CCL11, IL-2, IL-13, CXCL2 and Granulocyte Colony-Stimulating Factor) (AUC = 0.866, n = 150). Besga and colleagues (Besga et al., 2015) retrospectively compared late-onset BD to Alzheimer's disease, demonstrating consistent high performance (AUC = 0.86-0.96, n = 58) across SVM, CART, and RF algorithms, with IL-6 and TNF-a as the most relevant features across all models. However, attempts to differentiate BD from SZ proved more challenging, with Skorobogatov and colleagues (Skorobogatov et al., 2024) achieving only modest discrimination performance (AUC = 0.63, n = 692) and identifying no significant predictive features. An exception to these good-to-modest predictive performances came from childhood-onset psychiatric disorders. Ucuz and colleagues (Yildirim, 2020) achieved notably high classification performance (accuracy = 0.99, n = 211) in distinguishing early-onset SZ, BD, depressive disorder, and anorexia nervosa using an ANN-based multilevel perceptron model. The model incorporated 11 complete blood count-derived parameters, including cell counts, inflammatory ratios (NLR, PLR, MLR), and mean platelet volume. Interestingly, while individual markers achieved moderate performances (AUC = 0.60-0.64), the authors attributed the model's superior accuracy to the ANN's capacity for capturing complex, non-linear interactions when considering all parameters together. However, these results should be interpreted cautiously, as the study lacked internal and external validation and used only 3-folds cross-validation in a retrospective design, raising concerns about potential performance overestimation.

Only four studies validated their results in independent external cohorts. Sun L. and colleagues (Sun et al., 2024) applied SVM in a retrospective cohort to distinguish MDD from HC, identifying 3 significant proteins (TRPV2, ZNF713, and CTSL) f in a 10-fold CV framework with feature selection. While the model achieved an accuracy of 0.975 (n = 40) in the discovery cohort, performance dropped substantially in external validation (0.764, n = 100), suggesting potential overfitting or limited generalizability of the selected features. Liu and colleagues (Liu et al., 2022) applied DT in a retrospective design using whole blood count to distinguish between affective and psychotic disorders. The model achieved an AUC of 0.873 (n = 108) in the discovery cohort and 0.926 (n = 201) in the external cohort. However, none of the immune markers emerged as significant features. Crucially, data preprocessing and feature selection were performed before data partitioning in the discovery cohort, increasing the risk of data leakage. In contrast, Martinuzzi and colleagues (Martinuzzi et al., 2021) used Elastic Net regression in a retrospective design to distinguish between BD and MDD during a depressive episode. By implementing a 10-folds CV for hyperparameter tuning, 5 cytokines (IL-6, IL-10, IL-15, IL-27, and CXCL10) were associated with BD in the discovery cohort (AUC = 0.83, n = 462), with IL-10, IL-15, and IL-27 successfully replicated in external validation with maintained performance (AUC = 0.83, n = 133).

Diagnostic markers were also used for patients ‘stratification, aiming to identify subtypes within individual disorders or across diagnostic categories based on immune signature. Both supervised and unsupervised ML approaches were used. Supervised learning algorithms were implemented to classify SMI into known clinical subgroups. For example, Sánchez-Carro and colleagues (Sánchez-Carro et al., 2023) applied SVM in a prospective cohort to differentiate between melancholic and non-melancholic MDD subtypes, achieving an AUC of 0.76 (n = 171) based on CRP, TNF-*α* (along with triglycerides), with both immune markers emerging as significant predictors. In SZ, Malashenkova and colleagues (Malashenkova et al., 2023) used linear discriminant analysis in a retrospective design to distinguish major from simple neurocognitive psychosis relative to HC, identifying T helper17 axis and M1 macrophages as significant predictive markers (accuracy = 0.965, n = 99).

Unsupervised learning algorithms were employed to uncover data-driven subtypes. Two studies focused on identifying inflammation-based subtypes in SZ. Hoang and colleagues (Hoang et al., 2022) used PCA followed by hierarchical clustering in a prospective cohort, identifying two distinct inflammation-based subtypes among SZ patients and HC (silhouette score = 0.46, n = 80). Approximately 36% of patients fell into a high-inflammation subgroup characterized by significantly elevated pro-inflammatory cytokines (IL-1β, IL-6, IL-8, TNF-α), greater cortical thickness in paralimbic and temporal regions, and more pronounced alterations in structural brain network topology compared to the low-inflammation subgroup. No significant differences emerged between subgroups for symptom severity, cognitive performance, or illness duration. Similar patterns emerged in other studies. Lizano and colleagues (Lizano et al., 2021) identified in a retrospective cohort a high-inflammation subgroup (36% of psychotic patients vs 20% HC) with elevated CRP, IFN-*γ*, IL-1*β*, IL-8, IL-10, TNF-*α*, and vascular endothelial growth factor (VEGF), achieving a silhouette score of 0.59 (n = 200). Zhang and colleagues (Zhang et al., 2023) further corroborated these findings in a retrospective cohort, identifying two clusters of psychotic patients (silhouette score = 0.55, n = 184), with approximately 30% in the high-inflammation subgroup showing pronounced deficits in inhibitory behavioural control and visual sensorimotor functions.

Taking a different approach, Lalousis and colleagues (Alexandros Lalousis et al., 2023) applied semi-supervised clustering of inflammatory markers, in a retrospective cohort, identifying five distinct subgroups: Low Inflammation, Elevated CRP, Classic Inflammation, Elevated IFN-γ, and Anti-Inflammatory. All subgroups showed reduced temporal and hippocampal grey matter volumes compared to HC, with the Classic Inflammation subgroup exhibiting the most widespread deficits. Importantly, two subgroups (Low Inflammation and Elevated CRP) were successfully replicated in an external independent cohort, although performance metrics were not available.

Sæther and colleagues (Sæther et al., 2023) adopted a comprehensive transdiagnostic approach in a retrospective cohort, using canonical correlation analysis (CCA) followed by hierarchical clustering (silhouette score of 0.59, n = 1403) with bootstrapped cluster stability assessment and permutation testing to identify immune-cognitive subgroups across both SZ and BD. This analysis revealed two distinct subgroups: a “high cognition–low immune dysregulation” subgroup mainly consisting of HC (74%), and a “low cognition–high immune dysregulation” subgroup predominantly composed by SMI patients (76% SZ and 55% BD). The high immune dysregulation subgroup showed significantly elevated levels of IL-18 system cytokines (IL-18, IL-18BP, IL-18R1, IL-18RAP), BD-2 and reduced soluble Vascular Cell Adhesion Molecule 1 (VCAM-1), marked impairments in verbal learning and psychomotor processing speed, greater symptom severity, lower global functioning, and higher daily antipsychotic doses. Notably, the two subgroups did not differ in age at onset or illness duration, suggesting this immune-cognitive pattern transcends illness stage.

We further examined the AUC for multimodal and unimodal approaches for diagnostic markers (Supplementary Material Table S7). Unimodal approaches yielded overall to an AUC of 0.802 - 1.000, while multimodal an AUC of 0.490 - 0.994.

### 3.4 Predictive markers

Twelve models used FDA BEST predictive markers to evaluate response to treatment, using mainly RF and SVM, and incorporating an average of 18.833 input features (SD = ± 27.108, range: 2-75). These models primarily focused on predicting response to treatment in MDD (n = 7, AUC = 0.590-0.944) and SZ (n = 2, AUC = 0.778-0.895). Elbakary and colleagues (Elbakary et al., 2025) used a prospective longitudinal cohort to assess if baseline inflammatory markers (NLR, CRP, MLR, and PLR) can predict SSRI treatment response in MDD after 12 weeks, with PLR resulting as significant predictor (AUC = 0.59, n = 94). He and colleagues (He et al., 2024) used a longitudinal retrospective design to measure response to antidepressant at baseline and after 8 weeks. From 440 cytokines, seven were included after feature selection (AUC = 0.944, n = 133), but none of the markers were significant. Moreover, feature scaling was performed prior to data partitioning, with possible data leakage. Only one study used SVM to identify treatment resistant depression in a prospective cohort based on peripheral blood markers, achieving an AUC of 0.65 (n = 171) and TNF-α, CRP, and triglycerides emerging as significant predictors (Sánchez-Carro et al., 2023). Yee JY and colleagues (Yee et al., 2025) used a prospective design to assess antipsychotic responsiveness in SZ, using a set of 75 cytokines as input variables. They distinguished between clozapine-responsive and clozapine-resistant groups (AUC = 0.884, n = 48), identifying five significant predictors (e.g. CCL25, Cystatin D (CST5), CD5, CD137 (TNFRSF9), MMP10).

Only one study from Guilloux and colleagues (Guilloux et al., 2015) validated their results in an external cohort. They used a prospective design to classify between remitters versus non remitters MDD after 12 weeks of citalopram antidepressant treatment, using peripheral genes expression in input. The genes IFITM3 and TIMP1 were significant in the discovery (accuracy = 0.762, n = 67) and in the external cohort (accuracy = 0.762, n = 63) Although the model was properly implemented using both Nested and LOOCV for internal validation, the small sample size in both cohorts might limit the applicability of these results. Across all studies, unimodal approaches achieved AUC values of 0.75-0.884, while multimodal models showed a broader range of performances (AUC = 0.65-0.943, Table S7). This variability suggests that while multimodal approaches offer greater potential for improving predictive accuracy, their performance substantially depends on feature selection and model optimization.

### 3.5 Monitoring markers

Eight models employed FDA BEST monitoring markers to evaluate clinical state and symptom severity, incorporating an average of 5.875 input features per model (SD = ± 5.938, range:1-13). These models achieved variable performance (AUC = 0.713-0.950), though methodological and clinical heterogeneity and limited external validation constrain interpretation of these findings. Skorobogatov and colleagues (Skorobogatov et al., 2024) investigated disease state retrospectively in both SZ and BD, distinguishing between acutely symptomatic and stable patients, using several cytokines (e.g. IL-6, IL-8, IFN-*γ*, TNF-*α*, IL-17) as well as metabolites from the tryptophan–kynurenine pathway. These models achieved AUC values of 0.713-0.838 (n = 692), without identifying any significant individual marker. Focusing on severity stages, Martínez-Cao and colleagues (Martínez-Cao et al., 2024) employed SVM to classify SZ patients across five cross-sectional severity stages based on the Clinical Global Impression-Severity scale (CGI-S). The model integrated 12 variables across five dimensions: psychopathology, clinical features, cognition, functioning and inflammatory biomarkers (PLR, NLR, MLR). While all the variables resulted as significant, the moderate performance (balanced accuracy = 0.698, n = 212), the retrospective design, and the absence of external validation limit the applicability of this multimodal approach in clinical practice. Only one study included external validation. Tao and colleagues (Tao et al., 2025) developed three ANN models to classify patients based on depression severity in a prospective cohort. Using SHAP analysis, IL-17 emerged as the primary and significant contributor after initially evaluating both cortisol and IL-17. The refined model using IL-17 as predictor demonstrated robust diagnostic performance in the discovery cohort, achieving an AUC of 0.95 (n = 100) for classifying HC, 0.87 (n = 41) for moderate depression (Hamilton Rating Scale for Depression [HAMD-17] scores 17–24), and 0.87 (n = 31) for severe depression (HAMD-17 scores >24). Crucially, the external validation in an independent cohort of medicated patients with ongoing depressive episodes showed variable performances. While the model achieved a perfect discrimination for HC (AUC = 1.00, n = 8) and replicated good performance for severe depression (AUC = 0.91, n = 38), the prediction of moderate depression declined to AUC = 0.70 (n = 21). For monitoring markers, multimodal achieved an AUC of 0.870 - 0.95, while unimodal yielded AUC of 0.713-0.838 (Table S7). However, these performances should be interpreted cautiously given the substantial lack of external validation, variable sample sizes, and lack of sufficient reporting of methodological strategies to mitigate overfitting.

### 3.6 Prognostic markers

Six models used FDA BEST prognostic markers to evaluate remission or symptoms evolution, incorporating an average of 75.2 input features per model (SD = *±* 53.058, range: 2-113). Prognostic markers were employed in three studies, achieving limited performance with balanced accuracies ranging from 0.479 to 0.64. Using a retrospective design, Donnelly and colleagues (Donnelly et al., 2025) applied Elastic Net regression models to predict symptoms evolution in young adults affected by MDD with high somatic symptoms or high anxiety symptoms. The model included 10 cytokines as input features (CCL11, IL-2, IL-4, IL-5, IL-6, IL-7, IL-8, IL-10, IL-13, and IFN-γ) and used 20 folds-Nested CV for hyperparameter tuning, achieving balanced accuracies of 0.479-0.580 (n = 309). No individual marker reached statistical significance. Liu and colleagues (Liu et al., 2025) developed two tree-based models (Extra Trees and Gradient Boosting Machines) to predict remission versus non-remission in MDD and BD following 12 sessions of rTMS (repetitive transcranial magnetic stimulation). Baseline CRP was identified as a significant predictor, though performance metrics were not reported. Although the model was implemented on a prospective cohort using adequate data partitioning, the small sample size of 68 participants limits the applicability of these findings. Crucially, none of these prognostic models implemented external validation, thus preventing the assessment of model's generalizability. Only in multimodal settings were implemented for prognostic markers, while performances in unimodal designs are lacking.

## 4 Discussion

This review is the first that comprehensively synthesizes ML application in immune biomarker research for SMI. According to the FDA BEST framework, immune biomarkers have been mainly applied for diagnostic purposes, achieving variable but sometimes high performance in case-control classification and differential diagnosis. In this case, the most frequently investigated and significant biomarkers included pro-inflammatory markers (IL-6, IL-8, TNF-α, IFN-γ, and CRP), along with the anti-inflammatory cytokine IL-10, identifying potential biomarkers for SMI. Predictive, monitoring, and prognostic applications are comparatively underrepresented and show more heterogeneous results. In general, these performances must be interpreted cautiously given the near-complete absence of external validation, with only 6 studies validating their findings in independent cohorts. Although previous meta-analytic evidence on the application of ML to immune biomarkers in SMI is scarce, the available findings align with previous pooled estimates for BD diagnosis with accuracies up to 90% (Colombo et al., 2022), while prediction of treatment response based on immune-related genes reaches approximately 70% performance (Monopoli et al., 2025; Lee et al., 2018). Notably, across reviewed studies, only a subset of the originally collected biomarkers served as input features, with most diagnostic and predictive models relying on markers already well established in the literature (e.g., IL-6, IL-10, TNF-*α*, IFN-*γ*, and CRP) (Köhler et al., 2017). A different picture emerged for prognosis, which incorporated larger panels of immune biomarkers, possibly reflecting uncertainty about which immune processes drive trajectories in SMI. However, the effective clinical utility of these biomarkers for this outcome remains unclear, as models with extensive feature sets (up to 75.2 features for prognostic markers) often showed modest performance and lacked significant individual predictors.

One of the advantages of ML relies in providing strategies to efficiently deal with high-dimensionality, such as feature selection from larger panels, and ensemble methods capable of capturing data-driven non-linear interactions. Nevertheless, algorithms specifically designed for modelling complex interactions, such as XGBoost or SVM with non-linear kernels, and feature selection or dimensionality reduction techniques were only sparsely applied also in studies with a wide number of input feature. Another advantage of ML is the possibility to efficiently manage different kind of data, allowing to investigate whether the integration of heterogeneous data sources in the same model results in improved predictive performances. However, across the reviewed studies, multimodal models did not consistently outperform unimodal approaches, with both showing comparable predictive performance. While this finding might suggest that unimodal models perform equally well, a potential alternative explanation is related to the type of information added to inflammatory marker in proposed multimodal models. Recent meta-analyses indicating that ML models using multimodal data usually achieve superior predictive performance compared to unimodal models in both differential diagnosis (Monopoli et al., 2025) and prediction of treatment response for SMI (Monopoli et al., 2025; Lee et al., 2018). However, this advantage has been primarily demonstrated when multimodal models incorporate neuroimaging or neurophysiological data as input features, which were rarely included in the studies reviewed here (which mainly combined immune markers with cognitive or clinical data). Given the significant role of structural and functional brain abnormalities in SMI, and potential effect of inflammation of these brain correlates (Benedetti and Vai, 2023) future research incorporating such data is necessary to fully evaluate the potential benefits of multimodal models.

Considered the used algorithms, we observed a predominance of supervised algorithms applied for prediction of diagnosis, clinical outcomes, or validation of existing diagnostic groupings, such as melancholic depression or treatment-resistant subgroups. In contrast, data-driven approaches, aimed at discovering novel immune-based subtypes, were markedly underrepresented, although most of unsupervised models revealed meaningful immune-driven subtypes within psychiatric populations. These applications have been more extensively explored in SZ, where inflammation-based subgroups with distinct neurobiological profiles emerged (Liu et al., 2022; Hoang et al., 2022). Notably, three studies (Lizano et al., 2021; Zhang et al., 2023; Sæther et al., 2023) used transdiagnostic approaches across psychotic disorders and SMI more broadly, converging on the identification of high-inflammation subgroups that cut across traditional diagnostic boundaries. These studies consistently identified that approximately 30-36% of patients clustered into high-inflammation subgroups characterized by elevated pro-inflammatory markers, structural brain alterations, and cognitive deficits. The convergence of these findings indicate that immune dysregulation and cognitive impairment may constitute a shared pathophysiological pathway affecting a substantial subgroup of patients across the SMI spectrum, independent of specific diagnostic categories, with important implications for biologically-informed treatment stratification and targeted interventions. In more general perspective, growing evidence in immunopsychiatry supports the existence of immune-driven subtypes (Wessa et al., 2026b), yet their systematic identification remains limited. Future ML applications should move beyond predominantly supervised, confirmatory approaches and instead prioritize unsupervised, data-driven strategies aimed at subtype discovery. The current imbalance toward classification over discovery highlights a key methodological gap and an important direction for future research.

Overall, ML applied to immune biomarker has thus mainly far functioned primarily as a proof-of-concept tool for diagnostic classification, rather than to generate clinically actionable insights for SMI. Although relatively few studies have focused on patient stratification, treatment response prediction, prognosis, or longitudinal monitoring, encouraging predictive performance has been observed, in some cases approaching 80% AUC. Nevertheless, these applications remain preliminary given the lack of external validation and insufficient methodological transparency. Finally, the extremely high performance reported in some models, with AUC or accuracy values exceeding 0.99, raises concerns about overfitting, particularly given the often absence of reporting complementary metrics such as sensitivity, specificity, or the lack of a replication of results through external validation. More broadly, in-sample performance estimates are inherently optimistic regardless of model's complexity, and even simple linear models with a single predictor show inflated performances compared to out-of-sample predictions. These optimistic estimates increase with the number of features and decrease with sample size, as we observed, meaning that predictive models with extensive feature sets but modest sample sizes are particularly susceptible to overestimated performances (Poldrack et al., 2020). Taken together, these results suggest that ML applied to immune biomarkers are still in early stages, with substantial methodological improvements needed before these approaches can reliably guide treatment decisions or personalized care.

In particular, consistent limitations were identified throughout the inflammation data pipeline, from biological sample collection and standardization to statistical processing and its final integration as input for computational algorithms. Immune biomarkers are particularly sensitive to biological and procedural variability, which may substantially influence downstream ML performance and limit cross-study comparability. In contrast to more stable data modalities, such as neuroimaging data, peripheral immune measures are affected by circadian rhythms, metabolic status, medication exposure, acute illness phase, and environmental factors (Agrawal et al., 2018; Pavlović et al., 2024). Fasting status and diurnal time of blood collection are especially relevant given the circadian regulation of cytokines and immune cell subsets, with pronounced 24-h oscillations in leukocyte populations and inflammatory mediators (Diks et al., 2019), however they were not consistently reported. Restricting sampling to a defined morning window and standardizing fasting conditions may reduce within- and between-subject variance unrelated to the clinical phenotype of interest (Jansen et al., 2026). Similarly, the interval between blood draw and processing, centrifugation protocols, storage temperature, and freeze–thaw cycles can selectively affect immune cell viability and soluble marker concentrations, including cytokines, proteins, miRNAs and circulating tumor DNA (Agrawal et al., 2018). Batch effects represent an additional source of systematic bias. When samples are processed across multiple runs, plates, or laboratories, randomization procedures and appropriate technical controls should be implemented at the experimental design stage (Johnson et al., 2007). Statistical correction approaches, such as empirical Bayes–based harmonization methods (e.g., ComBat), should be transparently reported and incorporated within cross-validation pipelines to avoid information leakage (Pavlović et al., 2024). In addition, preprocessing strategies applied prior to model training, including log-transformation, normalization, and scaling, require careful consideration due to the typically skewed distribution and heteroscedasticity of immune markers and other high-dimensional omics features (Prelaj et al., 2024). These steps should be justified based on data distributional properties and consistently embedded within resampling frameworks. Preprocessing steps were frequently performed before data partitioning, or outside the CV loop, allowing information from the test set to influence model training leading to possible data leakage and resulting in overoptimistic performance estimates. Failure to control or report biological parameters and preprocessing methods may result in technical variance being inadvertently captured as predictive signal by ML algorithms (Pavlović et al., 2024; Koelzer et al., 2019). Therefore, systematic documentation and, where feasible, harmonization of pre-analytical variables should be considered a methodological priority in future studies to enhance stability, transportability, and long-term predictive validity of immunological ML signatures in severe mental illness and other clinical contexts.

Furthermore, several methodological weaknesses in models' implementation emerged. The reporting of missing data was frequently inadequate. When addressed, missing values were commonly handled through case-wise exclusion, or replacement with the lower limit of detection. These approaches increase the risk of bias, as excluding incomplete samples reduces statistical power and may introduce systematic errors when missingness is outcome-related. This is particularly problematic in ML, as model performance depends heavily on data quality. Models trained on poorly imputed data may therefore show artificially inflated performance but limited robustness and reliability (Shadbahr et al., 2023). Sample size is particularly important for ML performance interpretation, as small samples can inflate performance estimates (Varoquaux, 2018). Most of the studies included fewer than 50 participants, far below the several hundred of observations recommended for stable predictive accuracies (Poldrack et al., 2020). Consistent with this, our results demonstrated higher AUC values in datasets with fewer than 50 individuals and in models built with fewer than 10 features, suggesting that inflated performance estimates in underpowered datasets are likely attributable to overfitting rather than true predictive ability. Class imbalance was another recurrent issue, with few studies applying mitigation strategies, potentially inflating model's performance while hiding inadequate performance in smaller but clinically relevant groups. Feature selection or dimensionality reduction were also frequently absent, even in studies with large feature sets, increasing model instability and susceptibility to overfitting. Information regarding CV and train-test splitting were inconsistently reported, limiting the assessment of model generalizability. Only a small number of studies employed nested cross-validation, which is essential for managing the bias-variance trade-off and obtaining reliable performance estimates in high-dimensional settings (Trevor Hastie and Tibshirani, 2009). To provide reliable predictive models, rigorous CV procedures are strongly recommended to prevent data leakage, prioritizing nested and k-folds CV over leave-one-out approaches (Poldrack et al., 2020; Varoquaux et al., 2017).

As previously acknowledged, a further critical limitation concerns the lack of external validation. Only a minority of studies evaluated their models in independent cohorts, substantially limiting generalizability and representing a major barrier to clinical translation. Notably, all models that underwent external validation showed modestly lower but still meaningful performance for the same markers (e.g. elevated CRP subtype in schizophrenia (Alexandros Lalousis et al., 2023) and IL-17 for monitoring MDD disease state (Tao et al., 2025)), suggesting that some findings may retain clinical relevance. Future studies should prioritize external validation in independent cohorts with transparent reporting of validation strategies and out-of-sample performances to provide realistic and accurate estimates of predictive validity. Beyond external validation, the predominance of retrospective analytic designs further restricts inference regarding real-world applicability. Only a minority of investigations employed dedicated prospective designs aimed at addressing clinically relevant diagnostic or prognostic questions, while most relied on retrospective analyses of pre-existing cohorts. Such approaches raise concerns regarding cohort comparability, selection bias, and contextual confounding. These issues are particularly salient for immunological markers, which are highly sensitive to collection conditions, pre-analytical conditions, clinical context, inflammatory comorbidity, medication exposure, and environmental influences, and therefore more vulnerable to systematic differences between cohorts. Performance indices alone (whether AUC and other values) are insufficient to establish clinical usefulness if derived from non-comparable cohorts or not representative context. For biomarkers intended to inform personalized medicine, evaluation within prospectively defined, clinically representative populations are essential.

A related and potentially more critical methodological limitation is that most included studies systematically excluded participants with chronic or inflammatory conditions, despite these being common comorbidities in SMI (Mudra Rakshasa-Loots et al., 2025). This exclusion is particularly problematic given the increasing evidence suggesting the existence of a subgroup of MDD characterised by immune-mediated depression, often accompanied by chronic inflammatory or autoimmune comorbidities, more severe symptomatology (Wessa et al., 2026a) and treatment resistance (Chin Fatt et al., 2023). Excluding these individuals likely biases findings on immune alterations in SMIs, limiting both their validity and applicability to real-world clinical populations. These factors, together with pipeline heterogeneity, incomplete reporting of ML performance metrics, and substantial variability in immune biomarker selection, may have affected model robustness, fairness, and reliability (Liu, 2025), thereby limiting their validity and applicability to real-world clinical populations. At the same time, they reduced comparability across studies and precluded a quantitative meta-analytic synthesis of ML performance.

## 5 Conclusion

The present work demonstrates that ML applications in immunopsychiatry are expanding, but remain methodologically inconsistent, limiting the current reliability of immune biomarkers in predicting SMI outcomes. This is largely due to pronounced heterogeneity in algorithm implementation, and methodological weaknesses which restrict the clinical relevance of current findings. In general, translating the application of immune-based ML models into clinical tools, in line with FDA BEST framework, requires focusing on vali-dated, biologically relevant markers with strong analytical performance, including reliability, reproducibility across labs, tests, populations, and robustness to pre-analytical variation. From the computational side, this translates into interpretable models that consistently adopt standardized immune panels with harmonized pre-analytical protocols (including controlled fasting status, standardized collection timing, and systematic batch-effect correction) and implement rigorous pipelines to identify meaningful patterns across diverse populations. From the clinical perspective, future research should prioritize the application of ML algorithms to immune markers to clinically relevant outcomes beyond diagnosis. Integrating multimodal data and replicating findings across independent cohorts will be essential for advancing precision psychiatry and enabling personalized, immune-informed care.

## Authors’ contributions statement

Rayan Slatni, RS: conceptualization, search strategy of the included studies, title/abstract screening, full text screening, data extraction, data curation, formal analysis, writing and editing. Federica Colombo, FC: conceptualization, search strategy of the included studies, title/abstract screening, full text screening, data extraction, writing, editing, and reviewing. Paolo Enrico, EC: full text screening, data extraction. Igor Branchi, IG: supervising the project. Livia De Picker, LDP: reviewing, providing resources, and supervising the project. Benedetta Vai, BV: reviewing and supervising the project.

## Declaration of generative AI and AI-assisted technologies in the writing process

During the preparation of the final version of the manuscript the authors used GPT-5.2 (OpenAI) in order to improve fluency and readability. After using this tool, the authors reviewed and edited the content as needed and take full responsibility for the content of the publication.

## Funding

No funding.

## Conflict of interest

Unrelated to the submitted work, LDP reports speaker honoraria from Boehringer-Ingelheim and Janssen R&D. All the other authors declared no conflicts of interest.
