# Supplementary material for: Machine learning for immune biomarkers in severe mental illness: a systematic review
Source: Neurosci Appl. 2026 Apr 21;5:107003. doi: 10.1016/j.nsa.2026.107003 (PMC13126468; doi:10.1016/j.nsa.2026.107003)

**Machine Learning for Immune Biomarkers in Severe Mental Illness: a systematic review**

**Supplementary Materials**

**Table S1. Definition of the extracted variables.**

| **Variable** | **Meaning** |
| --- | --- |
| Study name | Study title |
| Authors | Authors of the study |
| Year | Publication year |
| Design | Cross-sectional, longitudinal, RCT etc. |
| Disorder / population | Psychiatry diagnosis |
| Diagnostic criteria | DSM or ICD |
| Infection / chronic inflammatory disease | Inclusion or exclusion criteria |
| Sample size | Sample size per each group |
| Sex |  |
| Age mean |  |
| Immune biomarkers | List of immune biomarkers measured (empty when the full list is not available) |
| Sample type | Whole blood, plasma, serum etc. |
| Analyte class | Cytokines, chemokines etc. |
| Assay platform | Immunoassay, multiplex etc. |
| Fasting | Fasting status at the time of blood collection |
| Time blood collection | Time when blood is collected |
| Blood processing | Blood processing and storage procedures |
| Batch effect | Control for any technical artefacts |
| Medication | If medication usage is reported and how |
| Acute phase | If the samples have been collected during acute phase with hospitalization for mental illness |
| Data transformation | Pre-processing steps that transform the data (e.g. normalization) |
| Missing data | % of missing data and imputation methods |
| Algorithms | List of algorithms executed that have immune markers are input feature |
| Learning paradigm | Supervised, unsupervised or semi-supervised |
| Input features | List of Immune biomarkers in input |
| Input features number | Number of immune biomarkers in input |
| Multimodal | (yes/no) |
| Input features COGNITION | List of cognitive features |
| Input features NEUROIMAGING | List of neuroimaging features |
| Input features BIOLOGICAL | List of biological features (not considering immune markers) |
| Input features CLINICAL | List of clinical features |
| Covariates | Covariate variable that are not input feature |
| Features selection | Features selection methods |
| Dimensionality reduction | Dimensionality reduction methods |
| Cross-validation | Cross-validation methods |
| Train test validation | (yes/no) |
| External validation | (yes/no) |
| Train test validation split | Train / test or train / test /validation |
| Hyperparameter tuning | Hyperparameter tuning methods |
| Algorithm objective | Diagnosis, subtyping, response, trajectory, severity, staging. |
| Model interpretability | Model interpretability methods |
| Class imbalance | (yes/no) |
| How handle class imbalance | Methods to mitigate the effect of class imbalance |
| Measures of performance | AUC, accuracy etc. |
| Algorithm performance | Report the performance results for each algorithm |
| Significant features | Features reported as significant in the study |
| Main findings | Report in few sentences the main finding of the study and additional comments. |

**Methods S1. Full search terms.**

**Pubmed**

("computational"[Title/Abstract] OR "machine learning"[Title/Abstract] OR

"deep learning"[Title/Abstract] OR "artificial intelligence"[Title/Abstract] OR

"AI"[Title/Abstract] OR "Explainable AI"[Title/Abstract] OR "XAI"[Title/Abstract] OR

"data-driven"[Title/Abstract] OR "clustering"[Title/Abstract] OR

"unsupervised learning"[Title/Abstract] OR "supervised learning"[Title/Abstract] OR

"predictive modeling"[Title/Abstract] OR "classification"[Title/Abstract] OR

"Bayesian model"[Title/Abstract] OR "dimensionality reduction"[Title/Abstract] OR

"multivariate analysis"[Title/Abstract] OR "network analysis"[Title/Abstract] OR

"predictive coding"[Title/Abstract] OR "systems biology"[Title/Abstract] OR

"precision"[Title/Abstract])

AND

( psychiatry[Title/Abstract] OR psychiatric[Title/Abstract] OR immunopsychiatry[Title/Abstract] OR   "severe mental illness"[Title/Abstract] OR "mental illness"[Title/Abstract] OR SMI[Title/Abstract] OR  psychos*[Title/Abstract] OR psychotic[Title/Abstract] OR "psychotic disorder*"[Title/Abstract] OR schizophrenia[Title/Abstract] OR  schizoaffective[Title/Abstract] OR bipolar[Title/Abstract] OR "major depressive disorder"[Title/Abstract] OR  MDD[Title/Abstract] OR "mood disorder*"[Title/Abstract] OR "affective disorder*"[Title/Abstract] OR  depression[Title/Abstract] OR biotyping[Title/Abstract] OR mania[Title/Abstract] OR manic[Title/Abstract] )

AND

( immune[Title/Abstract] OR immunophenotype[Title/Abstract] OR immunological[Title/Abstract] OR  inflammatory[Title/Abstract] OR inflammation*[Title/Abstract] OR "inflammatory marker*"[Title/Abstract] OR  biomarker*[Title/Abstract] OR "immune marker*"[Title/Abstract] OR immunomarker*[Title/Abstract] OR   cytokine*[Title/Abstract] OR chemokine*[Title/Abstract] OR interleukin*[Title/Abstract] OR "IL-6"[Title/Abstract] OR  "TNF-alpha"[Title/Abstract] OR CRP[Title/Abstract] OR "C-reactive protein"[Title/Abstract] OR  "immune profile*"[Title/Abstract] OR "immune signature*"[Title/Abstract] OR monocyte*[Title/Abstract] OR neutrophil*[Title/Abstract] OR "glial activation*"[Title/Abstract] OR microglia*[Title/Abstract] OR  "immune dysregulation*"[Title/Abstract] OR neuroinflammation*[Title/Abstract] OR  "acute phase protein*"[Title/Abstract] OR "immune cell phenotyping"[Title/Abstract] OR inflammasome[Title/Abstract] )

**Scopus**

( TITLE-ABS ( computational ) OR TITLE-ABS ( "machine learning" ) OR TITLE-ABS ( "deep learning" ) OR TITLE-ABS ( "artificial intelligence" ) OR TITLE-ABS ( AI ) OR TITLE-ABS ( "explainable AI" ) OR TITLE-ABS ( XAI ) OR TITLE-ABS ( "data-driven" ) OR TITLE-ABS ( clustering ) OR TITLE-ABS ( "unsupervised learning" ) OR TITLE-ABS ( "supervised learning" ) OR TITLE-ABS ( "predictive modeling" ) OR TITLE-ABS ( classification ) OR TITLE-ABS ( "bayesian model" ) OR TITLE-ABS ( "dimensionality reduction" ) OR TITLE-ABS ( "multivariate analysis" ) OR TITLE-ABS ( "network analysis" ) OR TITLE-ABS ( "predictive coding" ) OR TITLE-ABS ( "systems biology" ) OR TITLE-ABS ( precision ) ) AND ( TITLE ( psychiatry ) OR TITLE ( psychiatric ) OR TITLE ( immunopsychiatry ) OR TITLE ( "severe mental illness" ) OR TITLE ( "mental illness" ) OR TITLE ( SMI ) OR TITLE ( psychosis ) OR TITLE ( psychotic ) OR TITLE ( "psychotic disorder*" ) OR TITLE ( schizophrenia ) OR TITLE ( schizoaffective ) OR TITLE ( bipolar ) OR TITLE ( "major depressive disorder" ) OR TITLE ( MDD ) OR TITLE ( "mood disorder*" ) OR TITLE ( "affective disorder*" ) OR TITLE ( depression ) OR TITLE ( biotyping ) OR TITLE ( mania ) OR TITLE ( manic ) ) AND ( TITLE-ABS ( immune ) OR TITLE-ABS ( immuno ) OR TITLE-ABS ( immunophenotype ) OR TITLE-ABS ( immunological ) OR TITLE-ABS ( inflammatory ) OR TITLE-ABS ( inflammation* ) OR TITLE-ABS ( "inflammatory marker*" ) OR TITLE-ABS ( biomarker* ) OR TITLE-ABS ( "immune marker*" ) OR TITLE-ABS ( immunomarker* ) OR TITLE-ABS ( cytokine* ) OR TITLE-ABS ( chemokine* ) OR TITLE-ABS ( interleukin* ) OR TITLE-ABS ( IL-6 ) OR TITLE-ABS ( TNF-alpha ) OR TITLE-ABS ( CRP ) OR TITLE-ABS ( "C-reactive protein" ) OR TITLE-ABS ( "immune profile*" ) OR TITLE-ABS ( "immune signature*" ) OR TITLE-ABS ( monocyte* ) OR TITLE-ABS ( neutrophil* ) OR TITLE-ABS ( "glial activation*" ) OR TITLE-ABS ( microglia* ) OR TITLE-ABS ( "immune dysregulation*" ) OR TITLE-ABS ( neuroinflammation* ) TITLE-ABS ( biochemical* ) OR TITLE-ABS ( "acute phase protein*" ) OR TITLE-ABS ( "immune cell phenotyping" ) OR TITLE-ABS ( inflammasome ) )

**Web of Science**

TS=("machine learning" OR "deep learning" OR "support vector machine*" OR "random forest*" OR "gradient boosting" OR "neural network*" OR "predictive modeling" OR "explainable AI")

AND TI=(psychiatry OR psychiatric OR immunopsychiatry OR "severe mental illness" OR schizophrenia OR bipolar OR "major depressive disorder" OR depression OR psychosis OR "psychotic disorder*")

AND TS=("immune biomarker*" OR cytokine* OR "immune signature*" OR "inflammatory marker*" OR "immune dysregulation" OR microglia* OR "neuroinflammation" OR interleukin* OR "C-reactive protein" OR "immune profile*")

AND TS=(human OR humans OR patient* OR participant* OR subject*)

AND Article (Document Types) and English (Languages)

**PyscINFO**

(AB("machine learning" OR "deep learning" OR "support vector machine*" OR "random forest*" OR "gradient boosting" OR "neural network*" OR "predictive modeling" OR "explainable AI" OR "unsupervised learning" OR "supervised learning" OR classification OR clustering OR "bayesian model" OR "dimensionality reduction"))

AND (TI(schizophrenia OR bipolar OR "major depressive disorder" OR MDD OR psychosis OR "psychotic disorder*" OR "severe mental illness" OR SMI OR schizoaffective OR mania OR manic))

AND (AB(cytokine* OR chemokine* OR interleukin* OR "C-reactive protein" OR CRP OR TNF-alpha OR IL-6 OR microglia* OR "glial activation" OR "inflammatory marker*" OR "immune signature*" OR "immune profile*" OR "immune dysregulation" OR inflammasome)))

Filters: pubblicazioni accademiche, english, exclude animals

**Table S2. Most common Immune biomarkers per each SMI.**

| Immune biomarker | MDD | SZ | BD | N total |
| --- | --- | --- | --- | --- |
| IL-6 | 8 | 11 | 10 | 29 |
| IL-8 | 6 | 12 | 9 | 27 |
| TNF-α | 7 | 10 | 9 | 26 |
| IFN-γ | 5 | 9 | 7 | 21 |
| IL-10 | 7 | 9 | 8 | 24 |
| CRP | 6 | 7 | 5 | 18 |
| CCL11 | 6 | 5 | 4 | 15 |

Abbreviations: CCL, chemokine (C-C motif); CRP, C-reactive protein; BD, bipolar disorder; IFN, interferon; IL, interleukin; MDD, major depressive disorder; SZ, schizophrenia; TNF, tumor necrosis factor.

**Table S3. Most common Immune biomarkers for the four most used ML algorithms.**

| Immune biomarker | RF | SVM | Elastic Net | DT | XGBoost | N total |
| --- | --- | --- | --- | --- | --- | --- |
| IL-6 | 4 | 5 | 6 | 3 | 0 | 18 |
| IL-8 | 2 | 3 | 6 | 5 | 1 | 17 |
| TNF-α | 5 | 6 | 5 | 5 | 2 | 23 |
| IFN-γ | 2 | 4 | 5 | 3 | 1 | 15 |
| IL-10 | 3 | 6 | 5 | 3 | 1 | 18 |
| CRP | 7 | 4 | 2 | 1 | 2 | 16 |
| CCL11 | 3 | 3 | 5 | 1 | 1 | 13 |
| IL-7 | 1 | 2 | 6 | 1 | 0 | 9 |
| IL-16 | 1 | 0 | 5 | 0 | 0 | 6 |

Abbreviations: CCL, chemokine (C-C motif); CRP, C-reactive protein; DT, Decision Tree; IFN, interferon; IL, interleukin; RF, Random Forest; SVM, Support Vector Machine; TNF, tumor necrosis factor; XGBoost, Extreme Gradient Boosting.

**Table S4. Number of studies for each SMIs and the most for used ML algorithms.**

| SMI | RF | SVM | Elastic Net | DT | XGBoost |
| --- | --- | --- | --- | --- | --- |
| MDD | 10 | 7 | 5 | 1 | 4 |
| SZ | 7 | 6 | 2 | 4 | 2 |
| BD | 6 | 3 | 2 | 3 | 2 |

Abbreviations: bipolar disorder; DT, Decision Tree; MDD, major depressive disorder; RF, Random Forest; SMIs, severe mental illnesses; SVM, Support Vector Machine; SZ, schizophrenia; XGBoost, Extreme Gradient Boosting.

**Table S5.** **ML unsupervised algorithm performances per each model.**

| **Study author (s) (year)** | **Immune biomarkers** | **ML Algorithm** | **Algorithm objective** | **Diagnosis (sample size)** | **Silhouette score** | **Most relevant predictors** |
| --- | --- | --- | --- | --- | --- | --- |
| Lizano et al. (2021) | il-1β, il-6, il-8, il-10, IL12/IL23p40, IFNγ, TNF-α, TNF-β, CRP, Flt-1, VEGF, VEGFC, VEGFD, TGFβ1, C4a | HCL | subtyping | SZ + SZA + BD1 + HC (200) | 0,59 | CRP, IFNγ, IL-1β, IL-8, IL-10, TNF-α, VEGF |
| Shaojia Lu et al. (2013) | AgRP, b-FGF, BTC, GITR-L, I-TAC/CXCL11, IL-1β, IL-1 R1, MEC/CCL28, NT-4, TGF-β3, TECK/CCL25, TRAIL-R4, VEGF | HCL | subtyping | MDD + HC (65) | NA | AgRP, b-FGF, BTC, GITR-L, I-TAC, IL-1β, IL-1 R1, MEC, NT-4, TECK, TGF-β3, TRAIL-R4, VEGF |
| Lusi Zhang et al. (2023) | CRP, IL-6, C4a, IFNγ, IL-8, IL-10, VEGFD | HCL | subtyping | SZ + SZA + BD1 + HC (184) | 0,55 | CPR, C4q |
| SG Fillman et al. (2015) | IL-1β, IL-2, IL-6, IL-8, IL-18 | SPSS TwoStep Clustering | subtyping | SZ + HC (85) | NA | IL-6, IL-8, TNF-α |
| Dung Hoang et al. (2022) | il-1β, il-6, il-8, il-10, IL12/IL23p40, IFNγ, TNF-α, sFlt-1, bFGF, PlGF, VEGF | HCL | subtyping | FES + HC (80) | 0,46 | IL-1β, IL-6, IL-8, TNF-α |
| LS Sæther et al. (2022) | Inflammatory/Immune Canonical Variate | HCL | subtyping | SZ + BD1 + BD2 + PSY + SZA + HC (1403) | NA | IL-18 system, BD-2, VCAM-1 |
| Lalousis et al. (2023) | CRP, IFNγ, IL-10, IL-12, IL1-β, IL-2, IL-6, IL-8, TNF-α | HCL | subtyping | SZ + HC (1067) | 0,37 | IL-6, IL-8, CRP, IL-10 |

Abbreviation: BD, bipolar disorder; FES, first episode schizophrenia; HC, healthy controls; HCL, Hierarchical Clustering; NA, Not Available; PSY, psychotic; SVM, Support Vector Machine; SZ, schizophrenia disorders; SZA, schizoaffective disorder.

**Table S6.** **ML supervised regression algorithm performances per each model.**

| **Study author (s) (year)** | **Immune biomarkers** | **ML Algorithm** | **Algorithm objective** | **Diagnosis (sample size)** | **R2** | **RMSE** | **Most relevant predictors** |
| --- | --- | --- | --- | --- | --- | --- | --- |
| Lee Yena et al. (2021) | TNF-α, sTNFR1, il-1β, IL-2, IL-4, IL-6, IL-8, IL-10, IL-12 | DT | response | BD + HC (90) | 0,22 | 0,08 | IL-4, IL-6, IL-8, IL-1β, IL-2, IL-12, sTNFR1, IL-10, TNF-α, p-NFκB, p-FADD, p-IKKα/β, TNFR1, p-IRS1, p-p38, p-JNK |
| Popescu et al. (2025) | CRP, ESR | SVM | severity | SZ (70) | NA | NA | CRP |
| Foiselle et al. (2022) | IL-6, IL-7, IL-8, IL-12/IL-23p40, IL-15, IL-16, IL-17A, TNF-α | Elastic Net | subtyping | SZ + SZA (310) | NA | NA | IL-6, IL-7, IL-12/23 p40, IL16, TNF-α |

Abbreviation: BD, bipolar disorder; DT, Decision Tree; FES, first episode schizophrenia; HC, healthy controls; NA, Not Available; R2, coefficient of determination; RMSE,  root mean square error; SVM, Support Vector Machine; SZ, schizophrenia disorders; SZA, schizoaffective disorder.

**Table S7.** **Multimodal vs. Unimodal algorithm performance AUC (± standard error) for each algorithm objective.**

|  | Multimodal | n | Unimodal | n |
| --- | --- | --- | --- | --- |
| Diagnostic | 0.490 - 0.994 | 38 | 0.802 – 1.00 | 9 |
| Predictive | 0.650 - 0.943 | 5 | 0.75 - 0.884 | 3 |
| Monitoring | 0.870 - 0.950 | 3 | 0.713 - 0.838 | 3 |
| Prognostic | 0.73 - 0.863 | 2 | - | 0 |

Abbreviation: BD, bipolar disorder; FES, first episode schizophrenia; HC, healthy controls; HCL, Hierarchical Clustering; MDD, major depressive disorder; NA, Not Available; PSY, psychotic; SZ, schizophrenia disorders; SZA, schizoaffective disorder.

**Fig. S1. Shows the distribution of the immune biomarkers used as input features across studies**


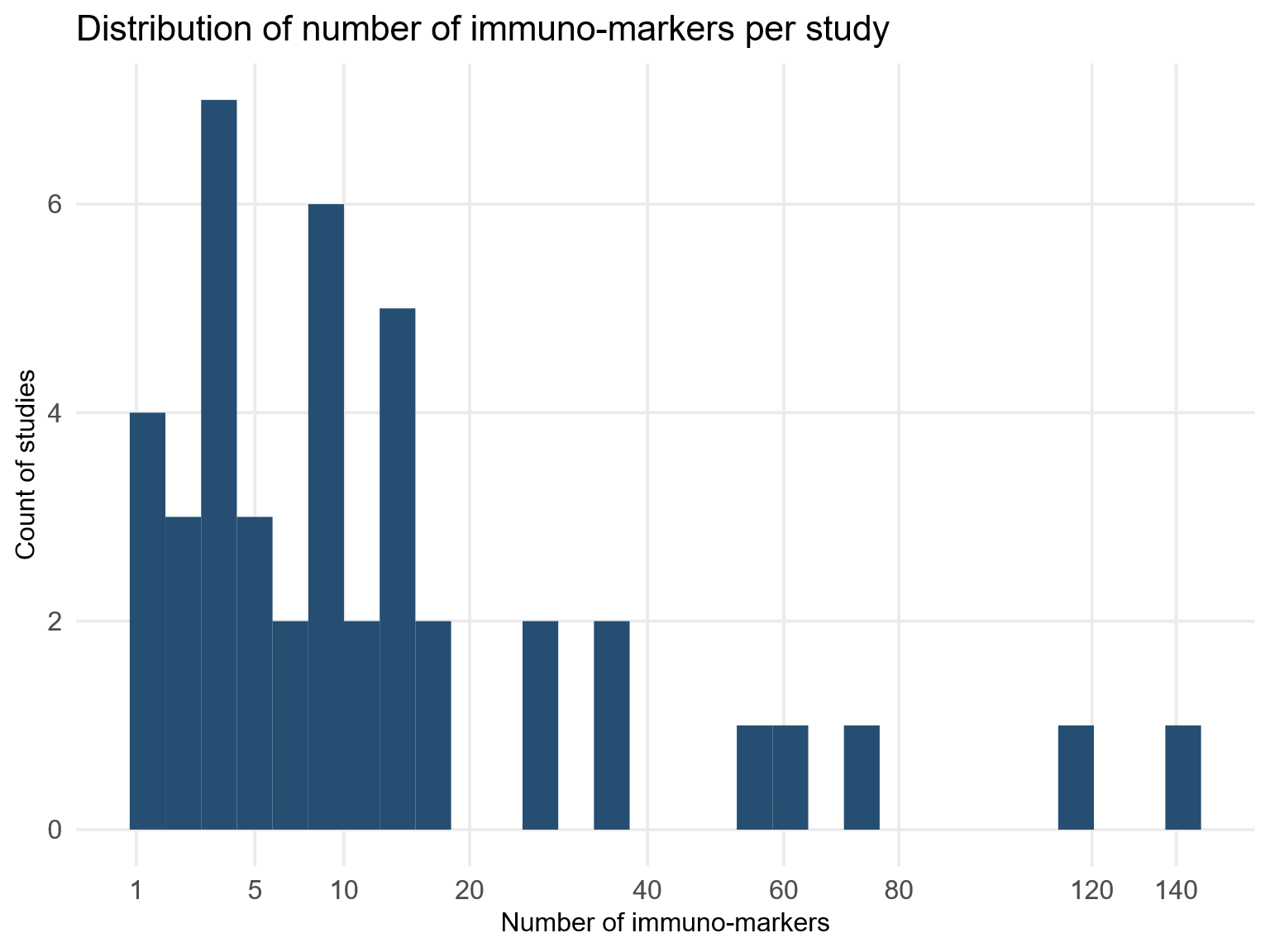


**Fig.S2. Barplot summarising the most used immune biomarkers across studies.**


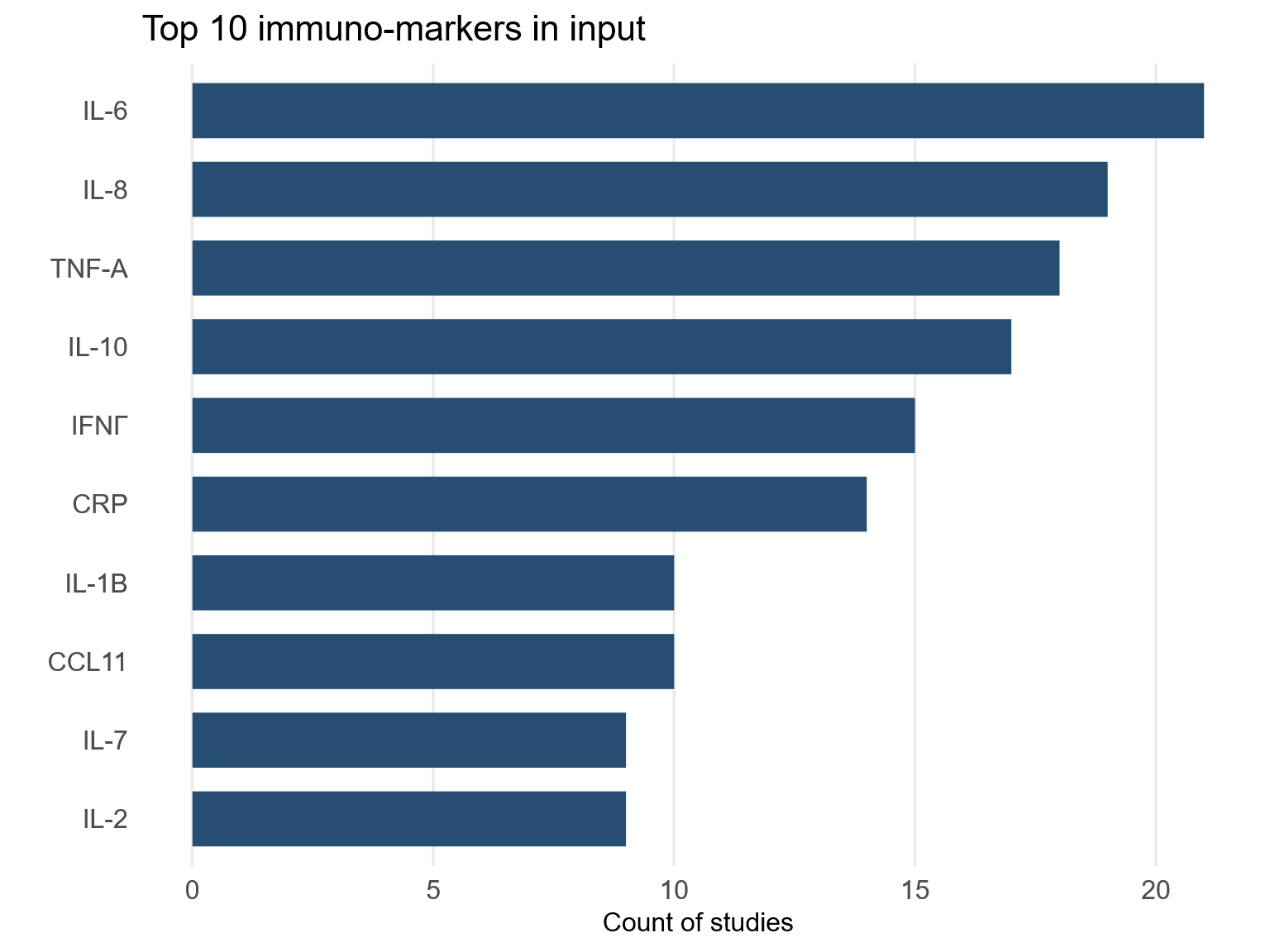


**Fig.S3. Barplot representing the ML algorithms across studies.**


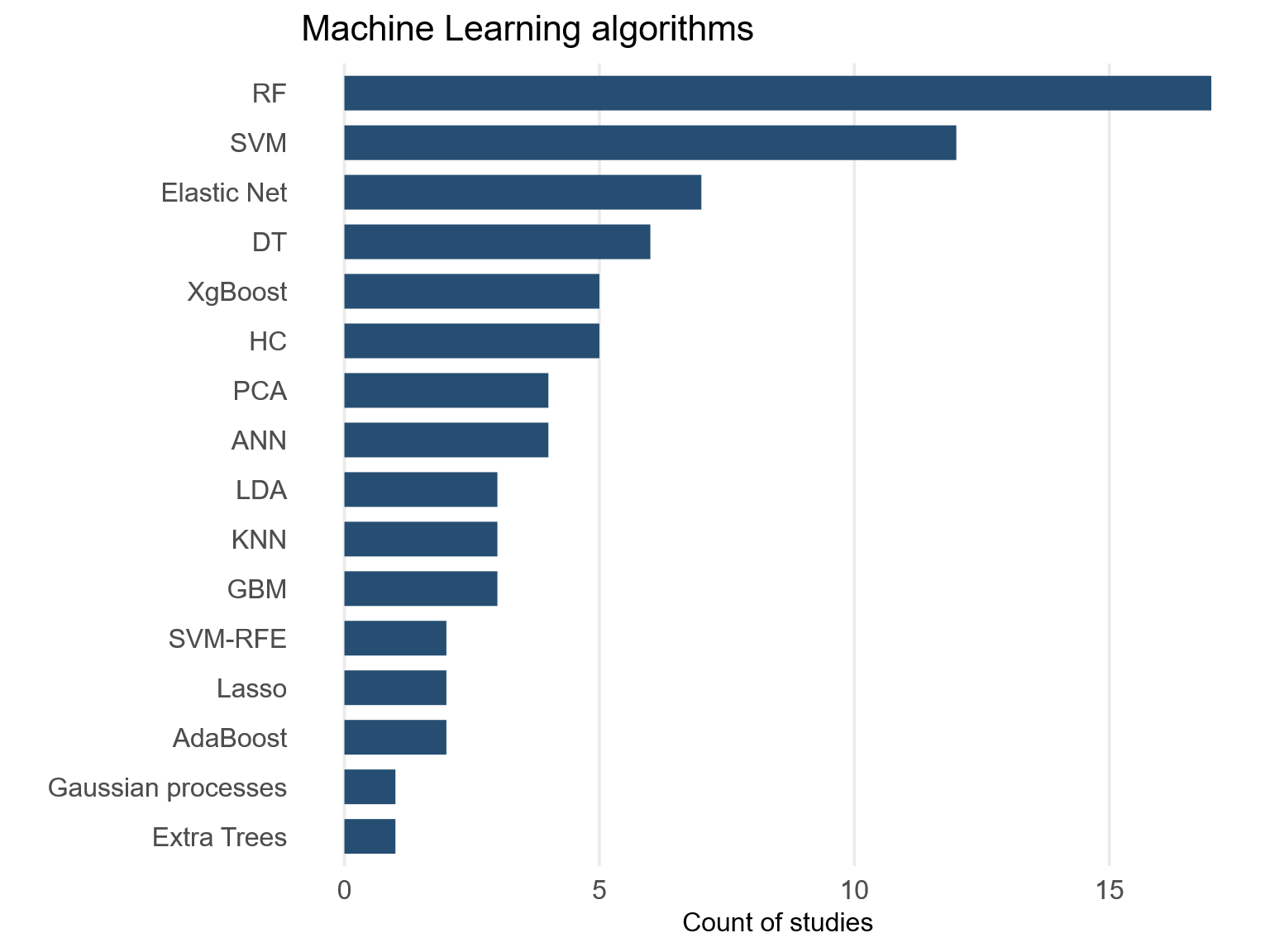

Supplement: Multimedia component 1 [file mmc1.docx]
